# LRRK2 regulates synaptic function through modulation of actin cytoskeletal dynamics

**DOI:** 10.7554/eLife.95987

**Published:** 2026-08-06

**Authors:** Giulia Tombesi, Shiva Kompella, Giulia Favetta, Chuyu Chen, Marta Ornaghi, Yibo Zhao, Ester Morosin, Martina Sevegnani, Adriano Lama, Antonella Marte, Ilaria Battisti, Lucia Iannotta, Nicoletta Plotegher, Laura Civiero, Franco Onofri, Britta J Eickholt, Giovanni Piccoli, Giorgio Arrigoni, Dayne Beccano-Kelly, Claudia Manzoni, Loukia Parisiadou, Elisa Greggio

**Affiliations:** 1 Department of Biology, University of Padova Padova Italy; 2 UK Dementia Research Institute, Cardiff University, Hadyn Ellis Building Cardiff United Kingdom; 3 School of Medicine, Cardiff University, Hadyn Ellis Building Cardiff United Kingdom; 4 Northwestern University Chicago United States; 5 Institute for Biochemistry, Charité Universitatsmedizin Berlin Berlin Germany; 6 Freie Universität Berlin Berlin Germany; 7 School of Pharmacy, University College London London United Kingdom; 8 CIBIO, University of Trento Trento Italy; 9 Department of Experimental Medicine, University of Genova Genova Italy; 10 IRCCS, Azienda Ospedaliera Metropolitana Genova Italy; 11 Department of Biomedical Sciences, University of Padova Padova Italy; 12 IRCCS San Camillo Hospital Venice Italy; Duke University United States; Stanford University School of Medicine United States

**Keywords:** Parkinson's disease, LRRK2, actin cytoskeleton, drebrin, BDNF, synapse, Human, Mouse

## Abstract

Parkinson’s disease (PD) is a multisystemic disorder that manifests through motor and non-motor symptoms. Motor dysfunction results from the degeneration of dopamine-producing neurons in the substantia nigra pars compacta. Increasing evidence suggests that synapse dysfunction precedes neuronal loss by years. Still, early synaptic alterations in PD remain poorly understood. Here, we integrate literature meta-analysis and multi-omics with biochemical, imaging, and electrophysiological measurements in *Lrrk2* mouse models and human iPSC-derived neurons lacking LRRK2. We demonstrate that brain-derived neurotrophic factor (BDNF) activates LRRK2 in differentiated SH-SY5Y cells and primary mouse neurons, reshaping the LRRK2 interactome toward a network of actin cytoskeleton-related proteins. Gene-ontology analyses of both literature-curated LRRK2 interactors and phospho-proteome from striatal tissues with elevated LRRK2 activity highlight synapse-actin remodeling as major affected pathways. We further observed that loss of LRRK2 impairs BDNF signaling and alters postsynaptic density architecture. Young *Lrrk2* knockout mice display structural alterations in dendritic protrusions, a phenotype that normalizes with age. In human iPSC-derived neurons, LRRK2 knockout affects maturation and BDNF-dependent regulation of spontaneous synaptic activity. Taken together, our study discloses a critical role of LRRK2 in BDNF-dependent synaptic modulation and identifies the synaptic actin cytoskeleton as a convergent site of LRRK2-associated pathophysiological processes in PD.

## Introduction

Synaptic damage and connectome dysfunction are emerging as early pathological events preceding neurodegeneration and the manifestation of clinical symptoms in multiple neurodegenerative disorders, including Parkinson’s disease (PD) (Fiala et al., 2002; Hong et al., 2016; Milnerwood and Raymond, 2010; Soukup et al., 2018; Picconi et al., 2012). PD is an age-related motor disorder for which no cure is available. The motor symptoms typically manifest as a consequence of the progressive loss of the dopaminergic neurons of the Substantia Nigra pars compacta (SNpc) projecting to the striatum (Kordower et al., 2013). The degeneration of the nigrostriatal projections precedes the loss of the dopaminergic cell bodies in the SNpc, and synaptic failure may be the triggering cause of axonal deterioration (Picconi et al., 2012; Bellucci et al., 2017). Pre- and postsynaptic alterations were described in PD animal models (Picconi et al., 2012). For example, PD pathology was reported as associated with an evident striatal spine loss that appeared to be correlated with the degree of dopamine denervation prevalently due to glutamate excitotoxicity in striatal neurons (Chidambaram et al., 2019; González-Rodríguez et al., 2021).

Accumulating evidence indicates that more than half of the causative genes and risk factors for PD have a function at the synapse (Leonard and Global Parkinson’s Genetics Program (GP2), 2025). Mutations in the *LRRK2* gene represent the most common cause of autosomal-dominant late-onset familial PD, and variations around the *LRRK2* locus increase lifetime risk for PD (Cookson, 2015). Pathogenic mutations are clustered into the enzymatic core of the LRRK2 protein, composed of a Roc/GTPase and a kinase domain, bridged by a COR scaffold (Greggio and Cookson, 2009; Marchand et al., 2020; Wallings et al., 2015). The most frequent mutation (G2019S) located in the kinase domain results in a protein with a gain of kinase activity, associated with increased cellular toxicity (Greggio and Cookson, 2009). Previous findings from our team and other laboratories suggested that LRRK2 sits at the crossroads between cytoskeletal dynamics and vesicular trafficking (Civiero et al., 2018; Dou et al., 2024; Boecker et al., 2021). LRRK2 activity seems to be modulated by interactions with a diverse array of cytoskeletal and vesicle-associated proteins, and it regulates membrane-trafficking events via phosphorylation of a subset of Rab GTPases, including RAB3, RAB8, RAB10, RAB12, RAB35, and RAB43 (reviewed in Civiero et al., 2018; Alessi and Pfeffer, 2024; Iannotta and Greggio, 2021). LRRK2 subcellular localization is dynamically controlled by phosphorylation/dephosphorylation of a cluster of N-terminal serine residues (e.g. Ser935 and Ser910), which determines 14-3-3 protein binding (Nichols et al., 2010). Phosphorylation of LRRK2 Ser935 and Ser910 is regulated by multiple kinases, including casein kinase 1 alpha (CK1a), IKKs, and protein kinase A (PKA) (Dzamko et al., 2012; Muda et al., 2014; Chia et al., 2014), and serves as a scaffold for 14-3-3 protein binding and LRRK2 subcellular localization (Nichols et al., 2010; Dzamko et al., 2010). In immune cells, extracellular signals such as Toll-like receptor 4 (TLR4) agonists stimulate LRRK2 Ser935 and Ser910 phosphorylation to positively modulate immune-related responses (Dzamko et al., 2012; Nazish et al., 2021). In contrast, the extracellular signals that orchestrate Ser935 phosphorylation enhancing LRRK2 activity in neurons are still unknown.

The ability of the neurons to relocate LRRK2 within or nearby the synapse could be particularly important as LRRK2 was suggested to provide the scaffold for the assembly of the signaling cascade effectors to sustain synaptic functions (Civiero et al., 2018; Maas et al., 2017; Cirnaru et al., 2014). In particular, LRRK2 has been shown to regulate synaptic vesicle cycling at the presynaptic site through interaction and phosphorylation of a panel of pre-synaptic proteins (Pischedda and Piccoli, 2021), such as NSF (Belluzzi et al., 2016), synapsin I (Marte et al., 2019), Endophilin A (Soukup et al., 2016), synaptojanin-1 (Cao et al., 2017), and DNAJC6/Auxilin (Nguyen and Krainc, 2018). Interestingly, recent literature supports a role for LRRK2 in the dendritic spine compartment, where it was suggested to influence AMPA receptor trafficking (Rassu et al., 2017; Gupta et al., 2024), spine morphology, and functionality in developing brains (Chen et al., 2020; Matikainen-Ankney et al., 2016; Parisiadou et al., 2014).

Here, starting from the observation that brain-derived neurotrophic factor (BDNF) stimulates LRRK2 Ser935 in neurons, we used unbiased protein-protein interaction (PPI) and phosphoproteomic investigations to disclose a novel function of LRRK2 in regulating actin-dependent synaptic dynamics in complementary neuronal models.

## Results

### BDNF treatment rapidly increases LRRK2 phosphorylation at Ser935

**Figure 1.**
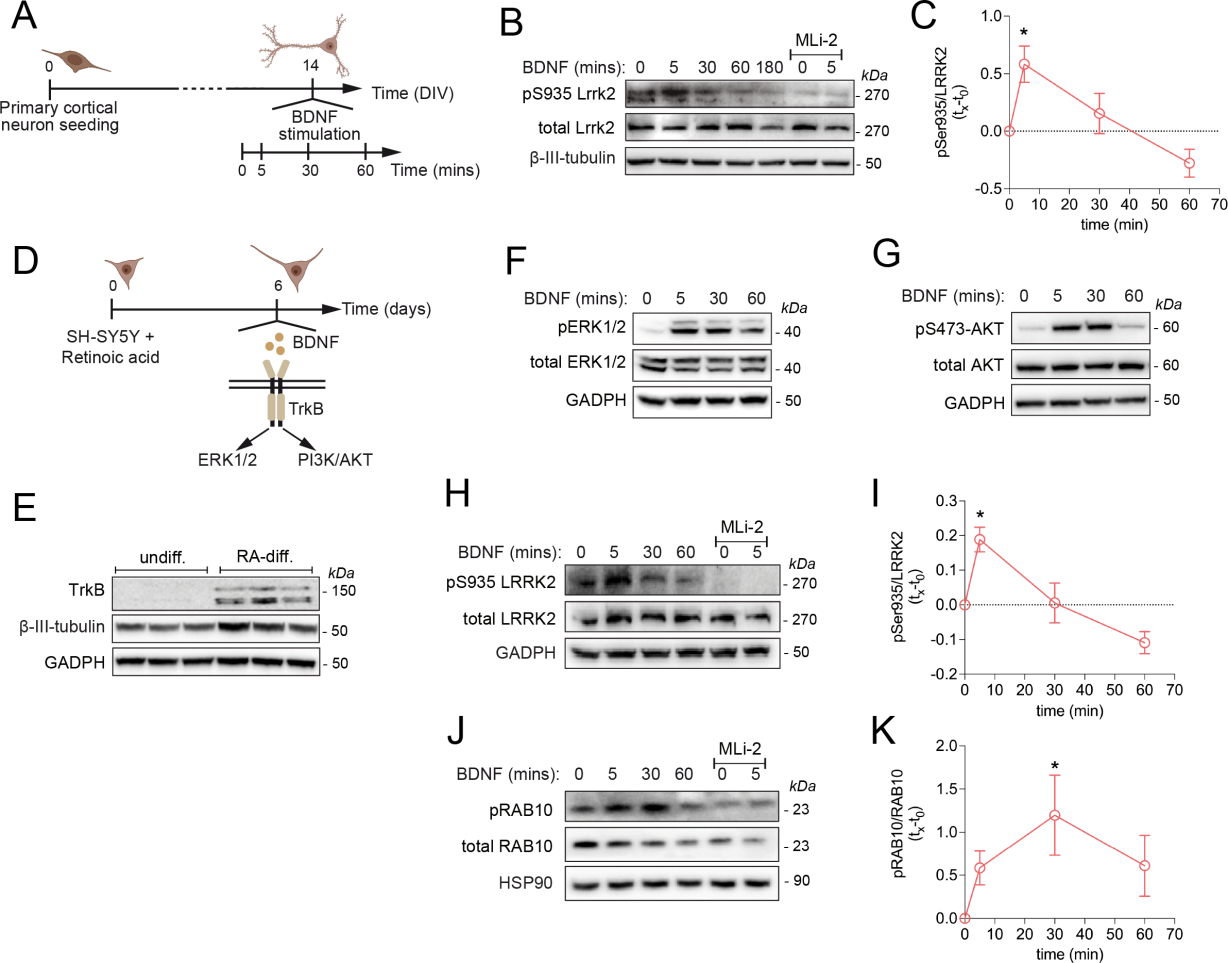
LRRK2 phosphorylation at Ser935 rapidly and transiently increases upon BDNF stimulation in primary mouse cortical neurons and differentiated SH-SY5Y cells. (**A**) Schematic representation of the experimental setting of (**B**). (**B**) Phospho-Ser935 and total LRRK2 protein levels of primary cortical neurons at DIV14 treated with 100 ng/mL BDNF for 0, 5, 30, 60, and 180 min. MLi-2 was used at 500 nM for 90 min to inhibit LRRK2 kinase activity. (**C**) Quantification of n=6 independent experiments of (**B**). One-way ANOVA (***p<0.001), Dunnett’s multiple comparison test (t=0 compared with different time points, *p<0.05 t=0 vs. t=5’). (**D**) Schematic representation of the experimental setting of (**F**) and (**G**). (**E**) TrkB protein levels in undifferentiated or retinoic acid-differentiated SH-SY5Y cells. (**F**) Phospho Thr202/185 ERK1/2, total ERK1/2, and (**G**) phospho Ser473 AKT, total AKT protein levels of retinoic-acid-differentiated SH-SY5Y cells stimulated with 100 ng/mL BDNF for 0, 5, 30, and 60 min. (**H**) Phospho-Ser935 and total LRRK2 protein levels of retinoic-acid-differentiated SH-SY5Y cells stimulated with 100 ng/mL BDNF for 0, 5, 30, and 60 min. MLi-2 was used at 500 nM for 90 min to inhibit LRRK2 kinase activity. (**I**) Quantification of (**H**) (n=4 independent experiments). One-way ANOVA (**p<0.01), Dunnett’s multiple comparison test (*p<0.05 t=0 vs. t=5’). (**J**) Phospho-T73 and total RAB10 protein levels of retinoic-acid-differentiated SH-SY5Y cells stimulated with 100 ng/mL BDNF for 0, 5, 30, and 60 min. MLi-2 was used at 500 nM for 90 min to inhibit LRRK2 kinase activity. (**K**) Quantification of (**J**) (n=4 independent experiments). One-way ANOVA (p>0.5), Dunnett’s multiple comparison test (*p<0.05 t=0 vs. t=30’). Figure 1—source data 1.Original western blot files for Figure 1. Figure 1—source data 2.PDF files containing original western blots for Figure 1, indicating the relevant bands.

Neurotrophic factors plastically shape neuronal activity by binding to their cognate receptor tyrosine kinases (Thoenen, 1995). BDNF is particularly important to modulate neurotransmission in the cortico-striatal circuitry (Baydyuk and Xu, 2014). This circuitry is relevant for PD and presents with the highest LRRK2 expression in comparison with the rest of the brain (Iannotta et al., 2020; Skelton et al., 2022). To address whether LRRK2 activity is stimulated by BDNF, we prepared primary mouse cortical neurons from C57BL/6 J wild-type mice and stimulated them with 100 ng/mL of BDNF at days in vitro 14 (DIV14) when synapses are fully functional (Figure 1A) and LRRK2 expression is detectable (Piccoli et al., 2011). Western blot analysis revealed a rapid and transient increase of Ser935 phosphorylation after BDNF treatment (Figure 1B and C). Of interest, BDNF failed to stimulate Ser935 phosphorylation when neurons were pretreated with the LRRK2 inhibitor MLi-2, indicating that either LRRK2 kinase activity controls the upstream kinases activated by BDNF or the binding with MLi-2, a type I inhibitor, locks the kinase in a conformation that favors dephosphorylation/hinders phosphorylation during the stimulus (Kluss et al., 2022).

**Figure 1—figure supplement 1.**
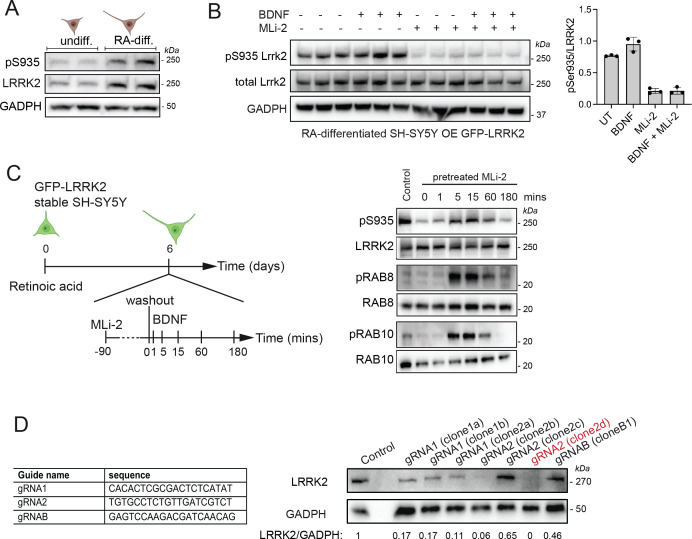
LRRK2 expression in naïve SH-SY5Y and characterization of SH-SY5Y stably expressing GFP-LRRK2. (**A**) Western blot analysis of undifferentiated versus differentiated naive SH-SY5Y and western blot with anti-phospho-Ser935 LRRK2 and total LRRK2 antibodies. (**B**) Cells stably overexpressing GFP-LRRK2 treated with 100 ng/mL BDNF for 5 min or with 500 nM MLi-2 for 90 min. (**C**) On the left, schematic representation of the experimental setting. On the right, BDNF stimulation of GFP-LRRK2 SH-SY5Y cells prior to MLi-2 (500 nM) treatment for 90 min (MLi-2 for 90’, washout, BDNF stimulation for 1’, 5’, 15’, 60’, and 180’). The timepoint 0’ corresponds to non-BDNF treated. Western blots were performed using anti-LRRK2 (phospho-Ser935 and total), anti-RAB10 (phospho Thr73 and total), and anti-RAB8 (pan-phospho-RABs and total RAB8). Figure 1—figure supplement 1—source data 1.Original western blot files for Figure 1—figure supplement 1. Figure 1—figure supplement 1—source data 2.PDF files containing original western blots for Figure 1—figure supplement 1, indicating the relevant bands.

To gain mechanistic insights into the effects of BDNF stimulation on LRRK2 function, we differentiated neuroblastoma SH-SY5Y cells with 10 µM retinoic acid (RA) treatment for 6 days (Encinas et al., 2000) and measured BDNF response by monitoring Tropomyosin receptor kinase B (TrkB) levels and phosphorylation of AKT and ERK1/2, two major downstream effector pathways of BDNF/TrkB signaling (Figure 1D; Numakawa et al., 2010). Expression of TrkB was undetectable in undifferentiated SH-SY5Y cells but was induced upon RA differentiation (Figure 1E). Accordingly, differentiated SH-SY5Y cells respond to 100 ng/mL BDNF stimulation, as evidenced by increased phosphorylation of ERK1/2 and AKT (Figure 1F–G). LRRK2 expression, low in undifferentiated cells, also increased upon differentiation (Figure 1—figure supplement 1A), indicating that this is a suitable neuronal model to study endogenous LRRK2. Notably, BDNF stimulation enhanced Ser935 phosphorylation with kinetics comparable with that observed in primary neurons (Figure 1H and I). To determine whether BDNF-induced Ser935 phosphorylation reflects LRRK2 kinase activation, we assessed phosphorylation of RAB10 at Thr73, an established LRRK2 substrate. As illustrated in Figure 1J and K, BDNF treatment significantly increased RAB10 phosphorylation, supporting the role of BDNF as a physiological activator of LRRK2.

### BDNF stimulates LRRK2 interaction with drebrin, an actin cytoskeletal-associated protein enriched at the postsynapse

To clarify the implications of BDNF-mediated LRRK2-activation on neuronal function, we used affinity purification coupled with mass spectrometry (AP-MS/MS). As the yields of endogenous LRRK2 purification were insufficient for AP-MS/MS analysis, we generated polyclonal SH-SY5Y cells stably expressing GFP-LRRK2 wild-type or GFP control. Notably, in this model, Ser935 phosphorylation showed only a modest increase upon BDNF treatment (Figure 1—figure supplement 1B) and prolonged stimulation led to a reduction of Ser935 phosphorylation below baseline levels (Figure 1B–C , and H–I). We hypothesized that this apparent limited response could be due to high basal levels of Ser935 phosphorylation, approaching a ceiling effect under resting conditions. This is consistent with previous MS analyses reporting that Ser935 is nearly fully phosphorylated (~100%) in the brain under basal conditions (Nirujogi et al., 2021). To test whether high basal phosphorylation masks BDNF-induced increases, we pretreated the cells with MLi-2 for 90 min to lower Ser935 phosphorylation. After inhibitor washout, cells were stimulated with BDNF at multiple time points. In these conditions, BDNF robustly increased both Ser935 and RAB phosphorylation, peaking at 5–15 min and returning to baseline by 60–180 min (Figure 1—figure supplement 1C). Notably, while the magnitude of pSer935 phosphorylation at the peak was similar to untreated controls – supporting the idea that Ser935 is near saturation under basal conditions – RAB phosphorylation levels significantly exceeded those of untreated cells. This indicates that, unlike Ser935, RAB phosphorylation remains far from saturated under basal conditions and serves as a more dynamic readout of LRRK2 kinase activity. These data further support BDNF as a physiological activator of LRRK2 and validate GFP-LRRK2 SH-SY5Y cells for interrogating stimulus-induced changes in LRRK2 signaling and protein interactions.

**Figure 2.**
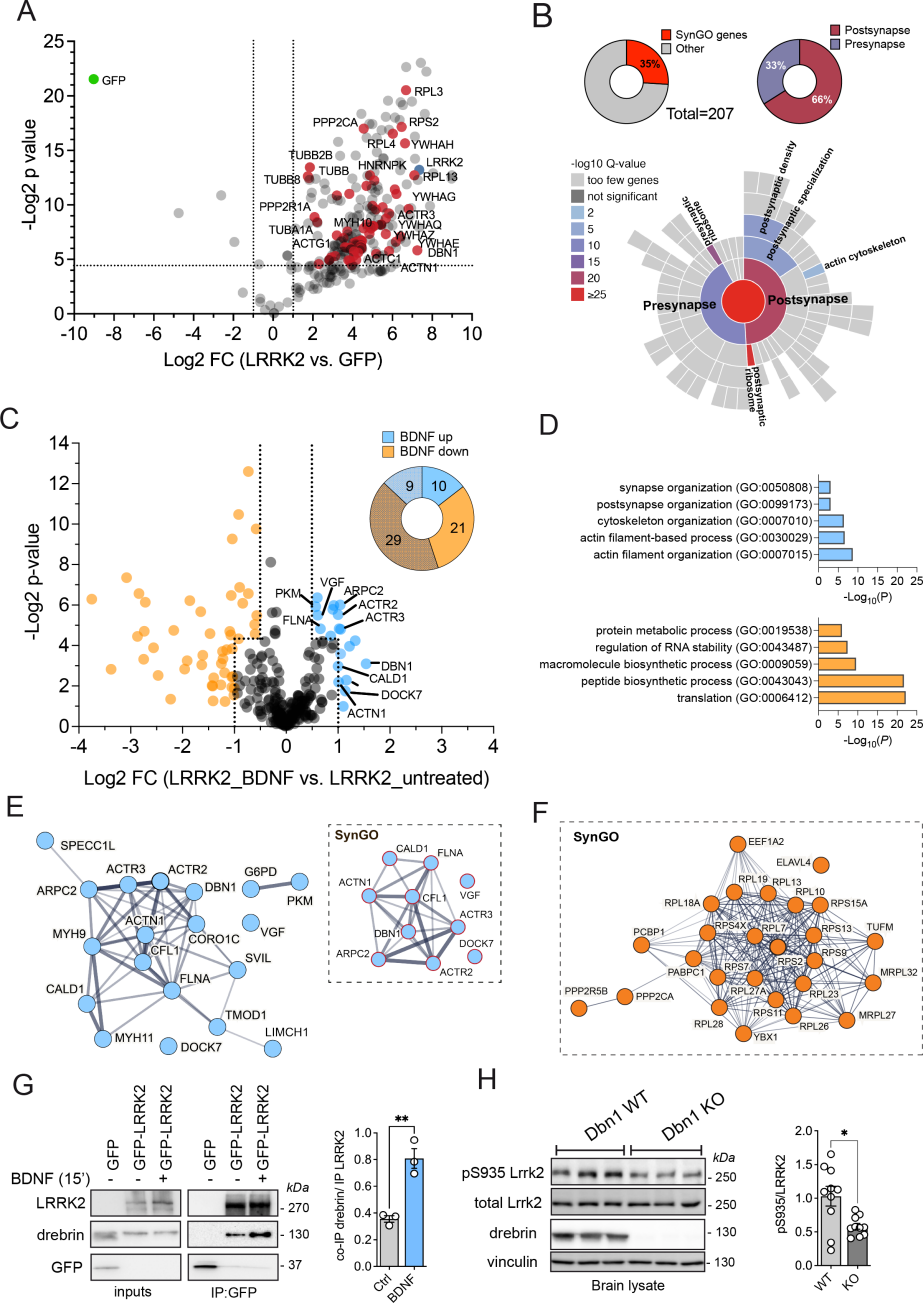
BDNF promotes LRRK2 interaction with post-synaptic actin cytoskeleton components. (**A**) Volcano plot of GFP-LRRK2 versus GFP enriched interactors from differentiated SH-SY5Y cells (n=6 replicates: n=3 independent experiments each with two technical replicates). Interactors that were considered for SynGO analysis have adjusted p-value <0.05 and FC >2 (red dots). (**B**) Donut charts with % of SynGO genes among the total 207 LRRK2 interactors (**A**) and with % of presynaptic and postsynaptic proteins among SynGO genes. SynGO-CC terms visualized with a sunburst plot showing significant categories (below). (**C**) Volcano plot of GFP-LRRK2+/-BDNF enriched interactors from differentiated SH-SY5Y cells (n=6 replicates: n=3 independent experiments each with two technical replicates). Interactors selected for pathway enrichment analysis fall into two categories: (**i**) adjusted p-value <0.05 and FC >0.5 or FC <–0.5, or (ii) |FC|>1 regardless of the p value. Proteins with increased interaction upon BDNF stimulation are blue-labeled, proteins with decreased interaction are orange-labeled. Donut chart showing the number of SynGO annotated genes versus non-synaptic proteins in BDNF-up versus BDNF-down interactors (top right). (**D**) G:profiler g:GOSt pathway enrichment analysis showing the top 5 enriched biological processes (BP) categories of BDNF-increased interactors (top, blue bar graph) versus BDNF-decreased interactors (bottom, orange bar graph). GO analysis was performed with g:Profiler on 04/14/2025 (version e112_eg59_p19_25aa4782, database updated on 03/02/2025); enriched terms determined using g:SCS correction and size terms set to <500 to increase specificity. (**E**) Protein-Protein Interaction Networks built with STRING (https://string-db.org/) of all BDNF(+) interactors and SynGO BDNF-increased interactors (inset) and (**F**) of SynGO BDNF-decreased interactors. (**G**) Validation of increased drebrin:LRRK2 interaction upon BDNF treatment (15 min) and quantification (one-sample t-test, p<0.01). Samples are the same analyzed in the three rounds of MS (**A**). (**H**) Western blot analysis of brain samples from WT and *Dbn1* (drebrin) KO mice (2-month-old) assessing Lrrk2 Ser935. Differences between the two genotypes have been evaluated using Student's t-test (significance *p<0.05 pSer935), n=10 animals per genotype. Figure 2—source data 1.Original western blots files for Figure 2. Figure 2—source data 2.PDF files containing original western blots for Figure 2, indicating the relevant bands.

Next, we used GFP-trap purification to isolate GFP or GFP-LRRK2 from RA-differentiated cells unstimulated or stimulated with BDNF for 15 min prior to MS analysis. The unstimulated LRRK2 interactome (GFP-LRRK2 vs. GFP) is large (207 significant hits) and consistent with previous computational analysis of LRRK2 PPIs (Figure 2A; Manzoni et al., 2015; Tomkins et al., 2018). In particular, we confirmed interactions with known LRRK2 binders including 14-3-3 proteins, cytoskeletal proteins, and protein translation factors (Supplementary file 1). Since BDNF is synaptically released and influences pre- and post-synaptic mechanisms, we used SynGO (Koopmans et al., 2019) to search for LRRK2 interactors that are enriched at the synapse. About one-third (35%) of LRRK2 PPI under unstimulated conditions are SynGO annotated genes; among them, 33% are presynaptic proteins and 66% are post-synaptic proteins (Figure 2B). The most significant enriched categories were pre- and post-synaptic ribosome and post-synaptic actin cytoskeleton (Figure 2B).

**Figure 2—figure supplement 1.**
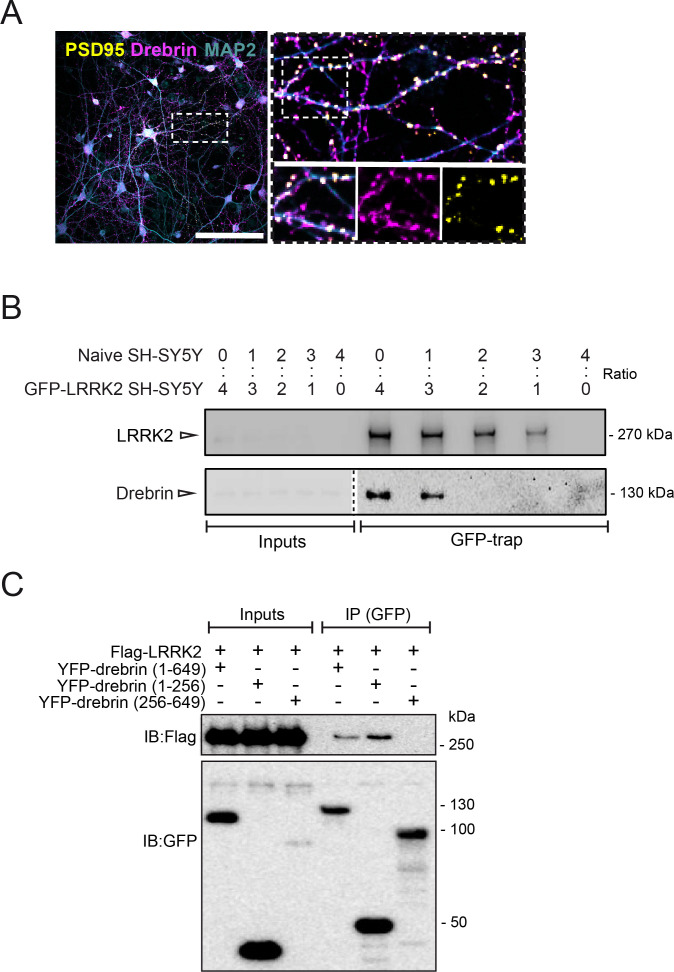
Western blot analysis validating the specificity of LRRK2-Drebrin interaction. (**A**) Confocal immunofluorescence of DIV14 primary neurons co-stained with MAP2 (neuronal marker), PSD95 (postsynaptic marker), and drebrin. Scale bar: 100 μm; magnifications scale bar: 10 μm. (**B**) To rule out that drebrin does not bind the resin but exclusively GFP-LRRK2 bound to anti-GFP nanobodies, GFP-LRRK2 was purified from cell lysates containing a mix of GFP-LRRK2 OE SH-SY5Y cells and naive SH-SY5Y cells at different ratios: 4:0, 3:1, 2:2, 1:3, 0:4 (OE:naïve). The levels of drebrin bound to GFP-LRRK2 diminish proportionally with the reduction of LRRK2 purified from the lysate, with a complete lack of signal in the eluate from the resin incubated with naive cells only (0:4 condition), confirming the specificity of drebrin binding to GFP-LRRK2. (**C**) Co-immunoprecipitation of 3xFlag-LRRK2 with YFP-drebrin domains (full-length, N-terminus, and C-terminus; amino acid boundaries are indicated in the figure). Drebrin constructs were immunoprecipitated with GFP-trap resin and co-precipitated LRRK2 visualized by western blot using anti-Flag antibodies. Figure 2—figure supplement 1—source data 1.Original western blots files for Figure 2—figure supplement 1. Figure 2—figure supplement 1—source data 2.PDF files containing original western blots for Figure 2—figure supplement 1, indicating the relevant bands.

We next compared unstimulated vs. BDNF-stimulated LRRK2 interactome. As shown in Figures 2C, 15 min of BDNF stimulation reshapes LRRK2 PPI, with a group of interactors increasing binding with LRRK2 (blue dots) and another group decreasing binding (orange dots). Inclusion criteria were: (i) p-value <0.05 and fold change (FC) >0.5 or FC <–0.5, or (ii) absolute fold change (|FC|)>1 regardless of the p value. In this way, we increased the discovery power for gene ontology analysis. SynGO gene ontology revealed that 10 out of 19 hits with increased binding after BDNF [BDNF(+)] and 21 out of 50 hits with decreased binding [BDNF(-)] are synaptic proteins (Figure 2C inset). Among synaptic interactors, the BDNF(+) binders are enriched in actin-cytoskeleton-related GO:BP categories while the BDNF(-) binders are enriched in protein translation GO:BP categories (Figure 2D). Furthermore, 80% of synaptic BDNF(+) LRRK2 binders (DBN1, ARPC2, ACTR2, ACTR3, CFL1, CALD1, ACTN1, FLNA) and 62% of synaptic BDNF(-) binders (RPS2, RPS4X, RPS7, RPS9, RPS11, RPS13, RPS15A, RPL13, RPL23, RPL26, RPL27A, TUFM, EEF1A2, PCBP1) form functional and physical interaction networks (Figure 2E–F).

To validate these findings, we performed western blot analysis on the AP samples analyzed by MS. We selected drebrin, encoded by *DBN1*, because it sits at the center of the BDNF(+) network (Figure 2E, inset SynGO) and, in DIV14 primary neurons, it colocalizes with PSD95, a postsynaptic marker (Figure 2—figure supplement 1A), confirming previous observations (Koganezawa et al., 2017). As predicted, BDNF treatment increases LRRK2:drebrin interaction by approximately twofold (Figure 2G and Figure 2—figure supplement 1B). To further characterize this interaction, we performed co-immunoprecipitation in HEK293T cells co-expressing Flag-LRRK2 and either full-length YFP-drebrin, its N-terminal (1–256 aa), or C-terminal (256–649 aa) fragments. These experiments confirmed a direct interaction between LRRK2 and drebrin, with the N-terminal domain appearing to mediate the majority of the binding (Figure 2—figure supplement 1C). Notably, this region contains both the ADF-H (actin-depolymerizing factor homology) domain and a coiled-coil motif known to associate with actin (Koganezawa et al., 2017; Shirao et al., 2017). Finally, we observed a significant decrease in Ser935 phosphorylation in *Dbn1* KO mouse brains (Figure 2H). This finding further strengthens the notion that drebrin forms a complex with LRRK2 that is important for its activity, for example upon BDNF stimulation.

We conclude that BDNF stimulation engages LRRK2 into actin cytoskeleton dynamics via its interaction with actin-regulatory proteins. While SH-SY5Y cells may only contain sparse synapses (Martin et al., 2022), the enrichment of this BDNF-dependent protein network at synapses indicates that this site might be relevant for LRRK2 function. Our findings also suggest that a pool of LRRK2 binds protein translation factors under unstimulated conditions, which redistributes toward actin-rich domains upon BDNF-TrkB signaling.

### Convergence on actin and synaptic processes in literature-curated LRRK2 interactome and LRRK2 G2019S striatal phospho-proteome

**Figure 3.**
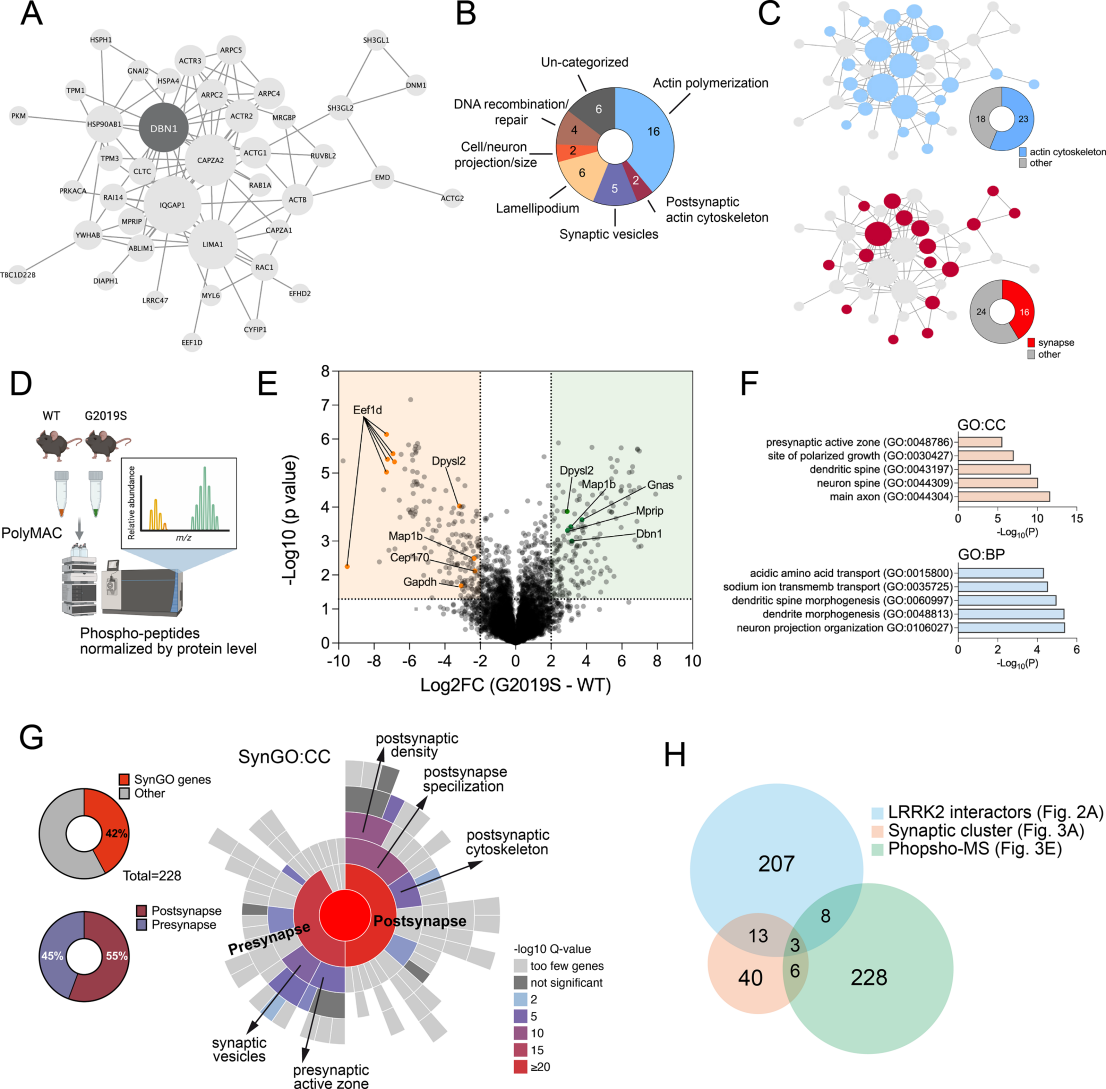
Convergence on actin and synaptic processes in literature-curated LRRK2 interactors and the LRRK2 G2019S striatal phospho-proteome. (**A**) Network graph showing one of the topological clusters extracted from the LRRK2 protein-protein interaction network. Proteins are represented as nodes while protein-protein interactions are represented as edges. Node size is proportional to the degree centrality: the larger the node, the higher the degree, the more interactions the node has in the network. (**B**) Gene Ontology biological-processes (GO-BPs) enrichment; the analysis was performed using g:Profiler, GO-BPs with term size >500 were discarded as general terms, and the remaining terms were clustered based on semantics using the keywords: Postsynaptic (Actin) Cytoskeleton, Lamellipodium, Synaptic Vesicle, Actin Polymerization/Nucleation, DNA Recombination/Repair, Cell/Neuron Projection. (**C**) Upper network graph as in (**A**) highlighting actin cytoskeletal proteins in blue and the proportion over the total (pie chart) and lower network graph as in (**A**) highlighting synaptic proteins in red and the proportion over the total (pie chart). (**D**) Experimental design of phospho-proteomic analysis comparing WT and G2019S knockin mouse striata. Phosphopeptide abundances were normalized to total protein levels. (**E**) Volcano plot of differentially enriched phosphopeptides, expressed as Log2 fold change (FC) between G2019S-KI versus WT. Highlighted peptides correspond to LRRK2 interactors discovered by MS in Figure 2. (**F**) GO enrichment analysis performed with g:Profiler (accessed on 01/23/2025; version e94_eg41_p11), filtering for GO terms with size <200 to avoid overly general categories. The top five most significant terms from the Cellular Component (CC) and Biological Process (BP) domains are shown. (**G**) Sunburst plot showing enriched SynGO CC categories and the percentage of SynGO genes among the differentially phosphorylated proteins. (**H**) Venn diagrams showing common and unique hits across the three datasets: GFP-LRRK2 interactomics, synaptic cluster, and phospho-MS.

We recently investigated the molecular environment surrounding LRRK2 specifically by constructing the PPI interactome around LRRK2 based on PPIs derived from peer-reviewed literature. After filtering the LRRK2 PPIs for reproducibility (keeping only those interactions that have been reported at least twice in literature or at least replicated with two different interaction detection methods), we obtained the interactions linking across the LRRK2 interactors thus effectively building the LRRK2 protein interaction network (LRRK2_net_), which describes the protein milieu around LRRK2 in the cellular context (see Zhao et al., 2024 for details). The LRRK2_net_ was topologically analyzed to identify the portions (clusters) of the network that are more densely connected locally in comparison with the overall connectivity of the entire network as these topological clusters are likely to represent functional units (Spirin and Mirny, 2003). Among the 11 topological clusters identified in Zhao et al., 2024, we further elaborated on one cluster in particular, containing 41 genes (Figure 3A) and found that it exhibited significantly GO-BP enriched terms into semantic categories related to: actin polymerization (16 GO-BP terms), synaptic vesicles (5 GO-BP terms), postsynaptic actin-cytoskeleton (2 GO-BP terms), lamellipodium (6 GO-BP terms), cell-neuron projection/size (2 GO-BP terms), DNA recombination/repair (4 GO-BP terms), and uncategorized (6 GO-BP terms; Figure 3B and Supplementary file 2). Out of the 41 proteins forming the cluster, 23 are proteins associated with the actin cytoskeleton, including the 4 larger nodes (where the dimension of the nodes is directly proportional to the node degree/connections within the network): DBN1/drebrin, CAPZA2, and LIMA, whose association with LRRK2 was identified in Meixner et al., 2011 and IQGAP1 identified in Liu et al., 2011; Figure 3C. Given the overrepresentation of synapse-related GO-BP terms, we further performed GO functional enrichment using SynGO and mapped 16 synaptic proteins within the cluster (Figure 3C). Remarkably, DBN1/drebrin is a key protein in the cluster, as it engages in multiple interactions (16 edges) and sits at the interface between the nodes involved in GO:BPs functions related to the actin cytoskeleton (blue-colored nodes) and to the synapse (red-colored nodes; Figure 3C and Figure 3—figure supplement 1).

**Figure 3—figure supplement 1.**
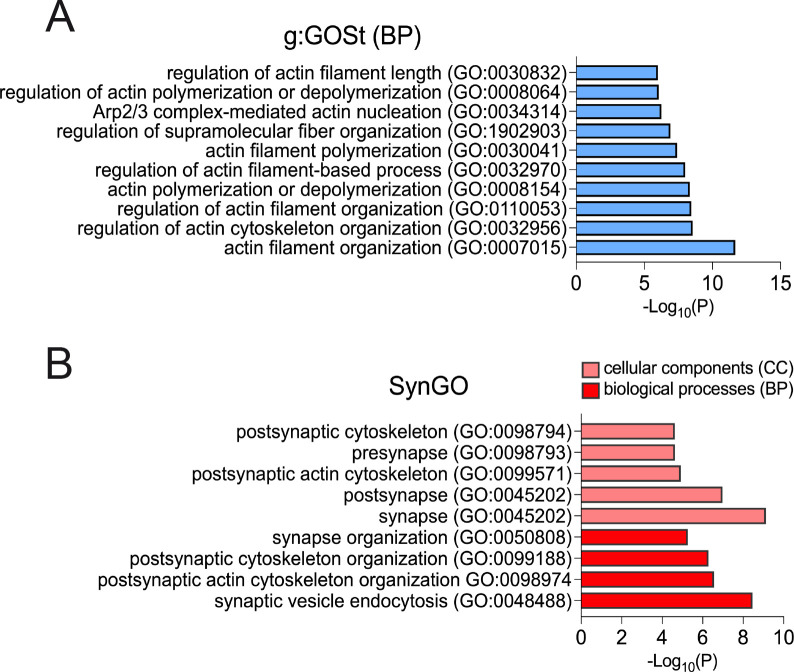
Actin Cytoskeleton and Synaptic Pathway Enrichment Analysis in literature-curated LRRK2 interactors. (**A**) Functional enrichment analysis; the analysis was performed using g:Profiler g:GOSt, proteins related to the actin cytoskeleton were manually identified and blue-colored, and the first 10 GO-BPs categories with term size <500 were graphed. (**B**) SynGO of enriched biological-processes and cellular components; synaptic proteins were red-colored, and significant terms in SynGO-BP and SynGO-CC categories were graphed.

**Figure 3—figure supplement 2.**
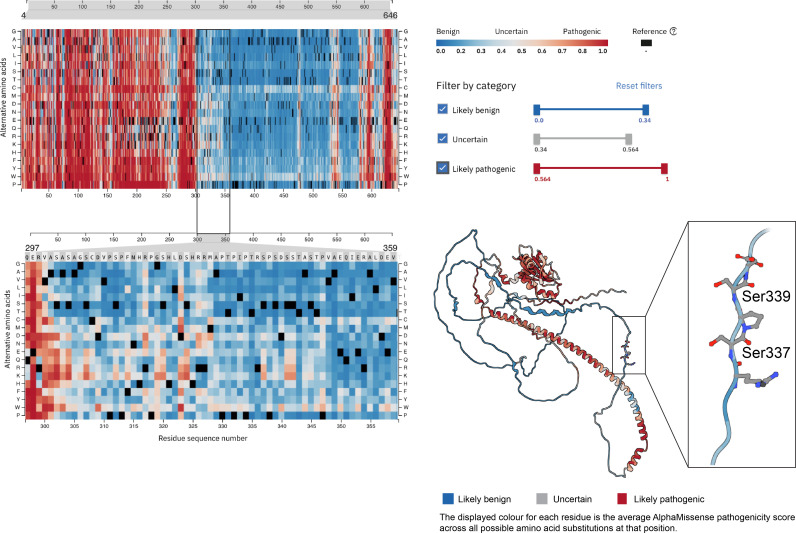
Analysis of the predicted amino acid pathogenicity in drebrin using AlphaMissense pathogenicity score. Amino acid change of S339 or its neighbor S337 is predicted to be benign, suggesting that phosphorylation at this site may play a regulatory function.

Given the multiple lines of evidence pointing to the synapse-actin couple as a key area for LRRK2 function, and based on the increased LRRK2 activity upon neurotrophin stimulation, we next asked whether LRRK2 kinase activity is also linked to synaptic function. To this aim, we performed an unbiased phospho-proteomic characterization of the striatum, using knockin mice expressing the hyperactive G2019S mutation to probe the contribution of kinase activity. We isolated total striatal proteins from WT and G2019S striata, enriched the phosphopeptides (Figure 3D) (also see Chen et al., 2025b), and normalized the log_2_ intensity of each phosphopeptide to the log_2_ intensity of its corresponding total protein. Comparing WT and G2019S samples, we identified 228 differentially regulated phosphopeptides (p-value < 0.05 and |Log_2_ fold change (FC)| > 2) (Figure 3E and Supplementary file 3). Gene ontology analysis of differentially phosphorylated proteins, using stringent term size (<200 genes), revealed enrichment of post-synaptic spines and presynaptic active zones (Figure 3F). A SynGO analysis confirmed enrichment of both pre- and postsynaptic categories, with particular high significance for terms related to the postsynaptic cytoskeleton (Figure 3G). Despite starting from whole striatal tissue – which contains a mix of different cellular populations – the strong synaptic enrichment of phosphorylation changes further supports the critical role of this compartment in LRRK2 brain function. By merging the three datasets (the LRRK2 interactome in SH-SY5Y cells, the literature-based synaptic/actin cluster and the phosphoproteomic dataset from G2019S striatum), we found three common hits: Dbn1/drebrin, Eef1d, and Mprip (Figure 3H). In particular, phosphorylation of drebrin at Ser339 was 3.7-fold higher in G2019S mice. This site is located in the N-terminal region of drebrin, which exhibited higher affinity for LRRK2 (Figure 3E, Figure 2—figure supplement 1C). To infer the potential physiological relevance of this phosphorylation, we examined its detection frequency in phosphoproteomic studies (67 observations, available here) and assessed its predicted pathogenicity using AlphaMissense (Cheng et al., 2023). Although the AlphaFold structural model has low confidence in this region of drebrin, amino acid substitutions at Ser339 are predicted to be non-pathogenic (Figure 3—figure supplement 2), supporting the notion that LRRK2-mediated phosphorylation at this site acts as a regulatory modification for drebrin activity.

### BDNF-mediated signaling is impaired in LRRK2 knockout neurons

**Figure 4.**
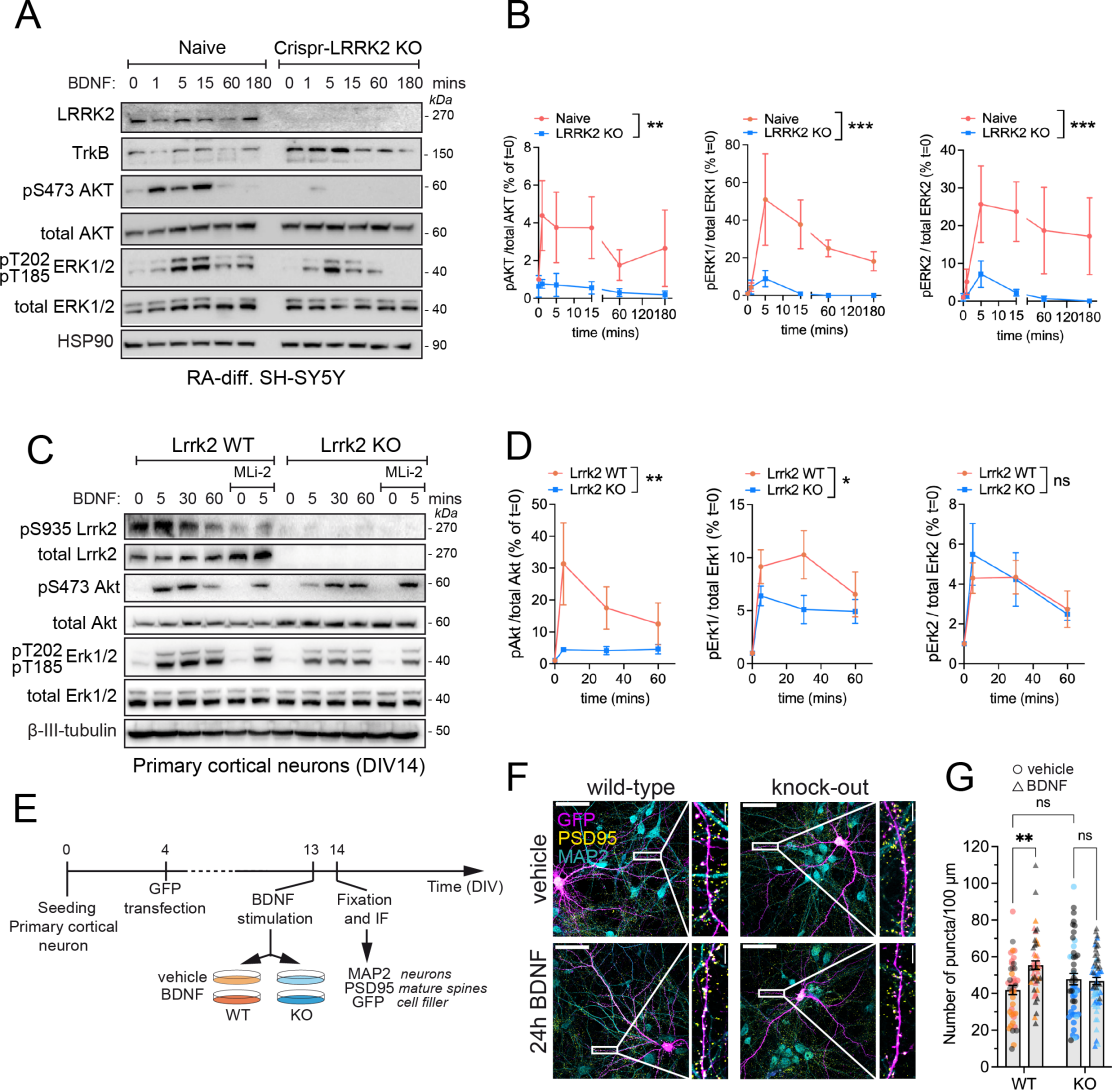
BDNF signaling is impaired in *Lrrk2* knockout neurons. (**A**) Six-day RA**-**differentiated SH-SY5Y naïve and CRISPR-LRRK2 KO cells stimulated with BDNF at different time points (0, 1, 5, 15, 60, and 180 min). Anti-TrkB antibodies were used to confirm expression of BDNF receptor. To compare BDNF-induced signaling in naïve vs LRRK2-KO cells, phosphorylated Akt (S473) and Erk1/2 (T185/T202) were evaluated. (**B**) Quantification of phosphorylated proteins shows from n=3 independent differentiation experiments. Two-way ANOVA; phospho-Akt: interaction p=0.7079, F (5, 24)=0.5896; genotype: **p=0.0014, F (1, 24)=13.07; time: p=0.5610, F (5, 24)=0.7994. Phospho-Erk1: interaction p=0.0783, F (5, 24)=2.284; genotype: ***p=0.0003, F (1, 24)=17.58; time: *p=0.0244, F (5, 24)=3.174. Phospho-Erk2: interaction: p=0.3725, F (5, 24)=1.128; genotype: ***p=0.0008 F (1, 24)=14.59; time: p=0.1356, F (5, 24)=1.879. (**C**) DIV14 primary cortical neurons from WT vs KO mice stimulated with BDNF at different time points (0, 5, 15, 30, and 60 min) in the presence or absence of LRRK2 inhibitor MLi-2 (90 min, 500 nM). To compare BDNF-induced signaling in WT vs KO neurons, phosphorylated Akt (S473) and Erk1/2 (T185/T202) were evaluated. Phosphorylated LRRK2 was assessed with pS935 antibodies. (**D**) Quantification of phosphorylated proteins shows from n=9 independent cultures. Two-way ANOVA; phospho-Akt: interaction p=0.1186, F (3, 63)=2.031; genotype: **p=0.0037, F (1, 63)=9.101; time: p=0.0342, F (3, 63)=3.069. Phospho-Erk1: interaction p=0.3256, F (3, 64)=1.177; genotype: *p=0.0201, F (1, 64)=5.680; time: ****p<0.0001, F (3, 64)=10.04. Phospho-Erk2: interaction *P*=0.8524, F (3, 62)=0.2622; genotype: p=0.7528, F (1, 62)=0.1001; time: ***p=0.0003, F (3, 62)=7.428. (**E**) Overview of the experimental workflow to induce spine formation/maturation. (**F**) Representative confocal images of primary cortical neurons stimulated with 100 ng/mL of BDNF or vehicle control for 24 hr. GFP has been transfected at DIV4 to fill the neuroplasm and visualize individual dendrites, MAP2 is a neuronal marker and PSD95 is a marker of mature spines. (**G**) Quantification of the number of PSD-positive puncta per unit of length (100 µm). Dots represent individual segments (*n*≅20 neurites from n=5–6 neurons per replicate), and colors define neuronal cultures prepared in different days from pulled pups (*n*≅8 pups per culture per genotype, n=3 independent cultures). Two-way ANOVA; interaction **p=0.0038, F (1, 191)=8.584; genotype: p=0.5833, F (1, 191)=0.3020; treatment: *p=0.0126, F (1, 191)=6.346. Šídák’s multiple comparisons test: vehicle vs. BDNF (WT) ***p=0.0005; vehicle vs. BDNF (KO) p=0.9440; Vehicle (WT) vs. vehicle (KO) p=0.3756. Figure 4—source data 1.Original western blot files for Figure 4. Figure 4—source data 2.PDF files containing original western blots for Figure 4, indicating the relevant bands.

Having collected multiple lines of evidence linking LRRK2 to actin cytoskeletal proteins enriched at the synapse, and given that BDNF – a neurotrophin known to promote synaptic plasticity – activates LRRK2 (Figure 1), we next asked whether loss of LRRK2 could affect BDNF signaling. To this end, we generated LRRK2 knockout (KO) SH-SY5Y cells with CRISPR/Cas9 editing (Figure 4—figure supplement 1), differentiated them with RA and assessed their response to BDNF stimulation. While application of BDNF increased AKT and ERK1/2 phosphorylation in naïve SH-SY5Y, as expected, LRRK2 KO cells exhibited a weaker response (Figure 4A–B). These results indicate that LRRK2 acts downstream of TrkB and upstream of AKT and ERK1/2, in line with its rapid and transient phosphorylation kinetics upon BDNF stimulation (Figure 1B–C).

**Figure 4—figure supplement 1.**
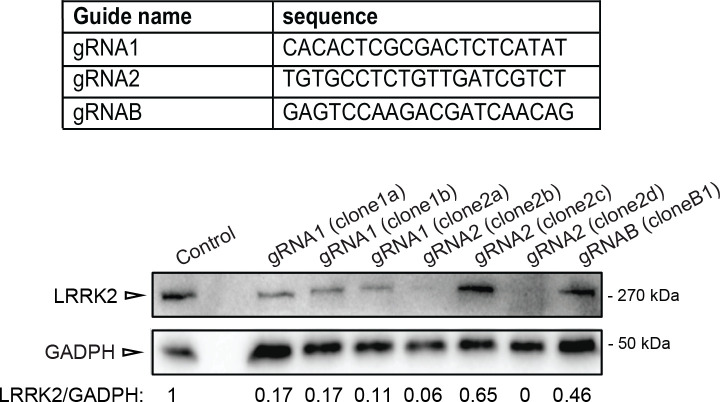
Western blot of different monoclonal populations of SH-SY5Y cells transfected with pSpCas9(BB)–2A-Puro (PX459) V2.0 vector (Addgene, Watertown, MA, US) for co-expression of Cas9 and gRNAs (gRNA1, gRNA2, and gRNAB) and subsequent single clone isolation and expansion. The band intensities corresponding to the total amount of endogenous LRRK2 were normalized to GAPDH, used as loading control. Figure 4—figure supplement 1—source data 1.Original western blot files for Figure 4—figure supplement 1. Figure 4—figure supplement 1—source data 2.PDF files containing original western blots for Figure 4—figure supplement 1, indicating the relevant bands.

Likewise, *Lrrk2* WT and KO primary cortical neurons at DIV14 were stimulated with BDNF at different time points. Similar to differentiated SH-SY5Y cells, *Lrrk2* WT neurons responded to BDNF by rapidly increasing Akt and Erk1/2 phosphorylation, while KO neurons exhibited a significantly reduced phosphorylation of Akt and Erk1 (Figure 4C–D).

Considering that (i) BDNF induces dendritic spine formation, maturation, and structural plasticity (Kellner et al., 2014; Zagrebelsky and Korte, 2014), (ii) BDNF(+) LRRK2 interactors are actin-related proteins enriched at the postsynapse (Figure 2), and (iii) loss of LRRK2 causes reduced phosphorylation of BDNF/TrkB downstream kinases AKT and ERK1/2 (Figure 4A–D), we evaluated BDNF-induced PSD95 sites in the presence or absence of LRRK2, as a potential process affected by LRRK2 deletion. Primary cortical neurons were transfected with GFP filler at DIV4 and treated with BDNF for 24 hr at DIV13 following previous protocols (Lai et al., 2012; Ji et al., 2005; Figure 4E). As shown in Figure 4F–G, BDNF treatment significantly increased the density of PSD95-positive puncta in WT cultures (p<0.001). In contrast, *Lrrk2* KO neurons did not exhibit increased PSD95 puncta upon BDNF. These findings further support a role for LRRK2 within the BDNF signaling cascade and show that the absence of LRRK2 impairs the remodeling of dendrite protrusions in response to BDNF stimulation.

### Morphological alterations of dendritic protrusions in young LRRK2 knockout mice

**Figure 5.**
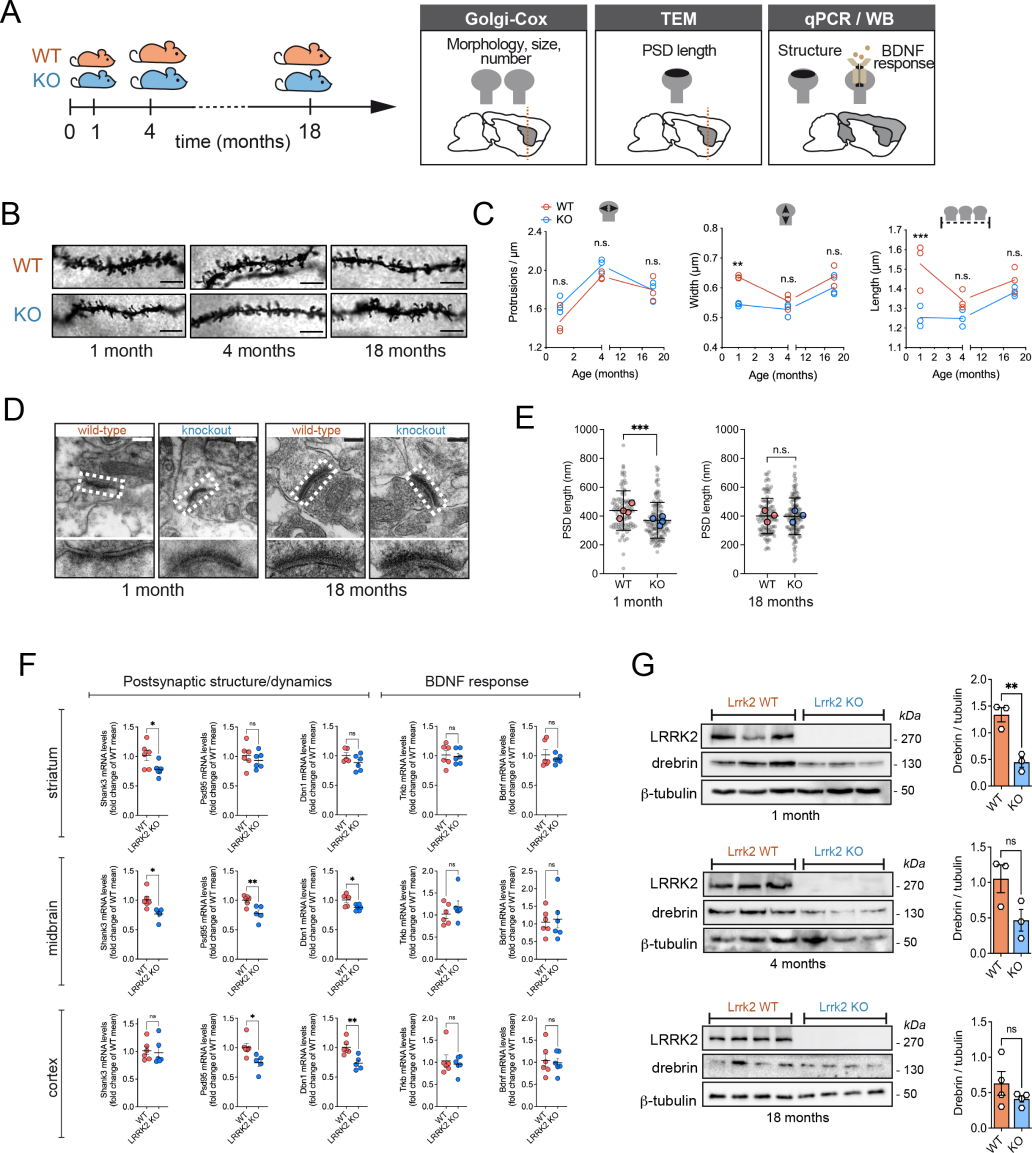
Postsynaptic structural changes in young *Lrrk2* knockout mice. (**A**) Overview of experimental design. (**B**) Representative images of neurite segments from Golgi-Cox stained neurons of dorsal striatum. Scale bar: 3 µm. (**C**) Quantification of average spine number (top), width (middle), and length (bottom) of n=3 animals per group (same segments analyzed in B). Statistical significance was determined by two-way ANOVA with Šídák’s multiple comparisons test. Number of protrusions: interaction p=0.3820, F (2, 12)=1.044; age: ****p<0.0001, F (2, 12)=27.12; genotype: p=0.0840, F (1, 12)=3.550; WT vs. KO (1 month) p=0.2013; WT vs. KO (4 months) p=0.5080; WT vs. KO (18 months) p>0.9999. Width: interaction p=0.0815, F (2, 12)=3.112; age: ***p=0.0004, F (2, 12)=16.14; genotype: ***p=0.0007, F (1, 12)=20.77; WT vs. KO (1 month) **p=0.0017; WT vs. KO (4 months) p=0.4602; WT vs. KO (18 months) p=0.p2489. Length: interaction *p=0.0345, F (2, 12)=4.514; age: *p=0.0203, F (2, 12)=5.488; genotype: ***p=0.0006, F (1, 12)=21.53; WT vs. KO (1 month), ***p=0.0008; WT vs. KO (4 months), p=0.2875; WT vs. KO (18 months), p=0.5948. (**D**) Representative transmission electron microscopy (TEM) micrographs of striatal synapses from 1-month and 18-month-old WT vs KO mouse brain slices. Scale bar: 200 nm. (**E**) Quantification of post-synaptic density (PSD) length. Graphs show the length of individual synapses (grey dots) and the average PSD length per animal (colored dots). Statistical significance has been calculated with Student’s t-test: 1-month-old mice (n=4 mice, 95 synapses WT, 118 synapses KO, ***p=0.0003); 18-month-old mice (n=3 mice, 108 synapses WT, 119 synapses KO, p=0.8502). (**F**) Quantitative PCR of *Bdnf, TrkB, Psd95, Shank3,* and *Dbn1* mRNA expression in striatum, cortex, and midbrain from n=6 *Lrrk2* WT and n=6 *Lrrk2* KO 1-month-old mice. Statistical significance has been calculated with Student’s t-test. (**G**) Western blot analysis of whole brain samples from the same Lrrk2 WT and KO mice where Golgi-Cox staining has been performed. The reduction in drebrin content is significant in Lrrk2 KO mice at 1 month of age. Differences between the two genotypes have been evaluated using Student’s t-test (significance **p<0.01), n=3–4 animals per genotype per age. Figure 5—source data 1.Original western blot files for Figure 5. Figure 5—source data 2.PDF files containing original western blots for Figure 5, indicating the relevant bands.

Based on in vitro results indicating that BDNF stimulates LRRK2 activity and promotes its recruitment to the actin-cytoskeleton, enhancing the number of PSD95-positive dendritic protrusions, we next investigated whether loss of *Lrrk2* results in dendritic spine defects in the mouse brain. During development, dendritic spines are highly dynamic, with a rate of extension and retraction that allows proper and accurate synapse formation and neuronal circuit assembly (Lendvai et al., 2000). By adulthood, dendritic spine formation, motility, and pruning decrease, reaching a relatively stable number of protrusions (Koleske, 2013). Since spine dynamics vary according to the stage of brain development, we performed a longitudinal analysis of young (1-month-old), adult (4-month-old), and aged (18-month-old) *Lrrk2* WT and KO mice in order to capture any age-dependent defect (Figure 5A). We focused on the dorsal striatum, a region highly enriched in SPNs that receives excitatory afferents from the cortex and dopaminergic modulation from the SNpc. This region is relevant in the prodromal stages of PD neurodegeneration (Surmeier et al., 2017) and expresses high levels of LRRK2 (Iannotta et al., 2020; Westerlund et al., 2008). Moreover, several studies showed a key role of LRRK2 in shaping the structure and function of excitatory synapses in the striatum (Chen et al., 2020; Matikainen-Ankney et al., 2016; Tong et al., 2009; Volta et al., 2017). We evaluated the number and morphology of dendritic spines using Golgi-Cox staining (Figure 5B). The total number of protrusions varied across ages, but there were no differences between the two genotypes (Figure 5C). Instead, the width and the length of the protrusions were reduced in 1-month-old *Lrrk2* KO striata with respect to WT. Specifically, the average neck height was 15% shorter and the average head width was 27% smaller, indicating that the protrusions are smaller in *Lrrk2* KO brains. No differences were observed at 4 and 18 months of age (Figure 5C). We then classified the morphology of the protrusions into four categories: filopodia and thin protrusions (immature spines) and mushroom and branched protrusions (mature spines). One-month-old *Lrrk2* KO animals exhibit a reduced number of filopodia and an increased quantity of thin protrusions, suggesting that loss of Lrrk2 may favor this transition. Instead, the proportion of mature spines (mushroom and branched) remained unaltered. No differences were observed in older mice (Figure 5—figure supplement 1).

**Figure 5—figure supplement 1.**
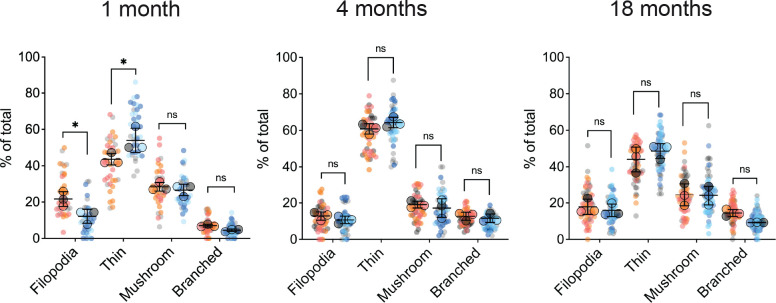
Morphological classification of protrusions into four classes (filopodia, thin, mushroom, branched) and quantified as % of the total number. Each dot represents one segment (n≥20 segments analyzed per animal; genotype: wild-type vs. *Lrrk2* KO; age: 1, 4, 18-month-old; n=3 mice per group) and error bars represent the mean ± SD of n=3 mice (color coded). Statistical significance was determined by two-way ANOVA with Šídák’s multiple comparisons test on mean values. One-month: interaction: **p=0.0017, F (3, 16)=8.018; genotype: p=0.5731, F (1, 16)=0.3309; class of protrusion: ****p<0.0001, F (3, 16)=158.4; filopodia WT vs. KO p=0.0201; thin WT vs. KO *p=0.0102; mushroom WT vs. KO p=0.9585; branched WT vs. KO p=0.8726. Four-months: interaction: p=0.3178, F (3, 16)=1.271; genotype: p=0.7300, F (1, 16)=0.1233; class of protrusion: ****p<0.0001, F (3, 16)=438.9; filopodia WT vs. KO p=0.8478; thin WT vs. KO p=0.4921; mushroom WT vs. KO p=0.8882; branched WT vs. KO p=0.9858. Eighteen-months: interaction: p=0.3237, F (3, 16)=1.253; genotype: p=0.6880, F (1, 16)=0.1672; class of protrusion: ****p<0.0001, F (3, 16)=69.67; filopodia WT vs. KO p=0.9756; thin WT vs. KO p=0.6413; mushroom WT vs. KO p>0.9999; branched WT vs. KO p=0.5290.

Since the overall width/height of *Lrrk2* KO protrusions is reduced (Figure 5C), we next analyzed the postsynaptic ultrastructure using electron microscopy. To increase the experimental power, we used a separate group of animals. We measured the postsynaptic density (PSD) length as it directly correlates with the amount of postsynaptic receptors and signaling proteins, thus providing an indication of the synaptic strength (Figure 5D). PSD is significantly shorter in 1-month-old *Lrrk2* KO animals compared to WT (p<0.001), while no differences in PSD length are observed in 18-month-old KO mice (Figure 5E). Taken all these data together, we conclude that subtle defects in the postsynaptic density are present in the striatum of postnatal *Lrrk2* KO mice, but these abnormalities appear to normalize with age.

To assess whether the loss of *Lrrk2* in young mice leads to reduced dendritic protrusion size and postsynaptic density through alterations in BDNF-TrkB signaling or postsynaptic scaffold expression, we measured transcript levels of *Bdnf*, *TrkB*, *Dbn1, Psd95,* and *Shank3*. Neither *Bdnf* nor *TrkB* expression is altered in the striatum, cortex, or midbrain of 1-month-old *Lrrk2* KO as compared to WT mice (Figure 5F), supporting a mechanism whereby LRRK2 acts downstream of BDNF signaling. Instead, *Dbn1*, *Psd95,* and *Shank3* expression levels are reduced in *Lrrk2* KO mice across most brain regions analyzed, with the most pronounced decrease observed in the midbrain (Figure 5F).

Given (i) the central role of *Dbn1*/drebrin in the LRRK2 synaptic network (Figures 2 and 3), (ii) its key function as an actin regulatory protein at the synapse, and (iii) its expression highly enriched at dendritic spines (Figure 2—figure supplement 1), we assessed drebrin protein levels at the three stages of life in whole brain lysates. In agreement with the qPCR results, drebrin levels are significantly lower in 1-month-old *Lrrk2* KO mice but not in older animals (Figure 5G), further linking altered spine maturation with improper actin-dynamics in the absence of LRRK2.

Taken together, our results indicate that LRRK2 influences dendritic spine structural dynamics during brain development.

### LRRK2 knockout in human iPSC-derived neurons affects maturation and BDNF-dependent regulation of spontaneous synaptic activity

**Figure 6.**
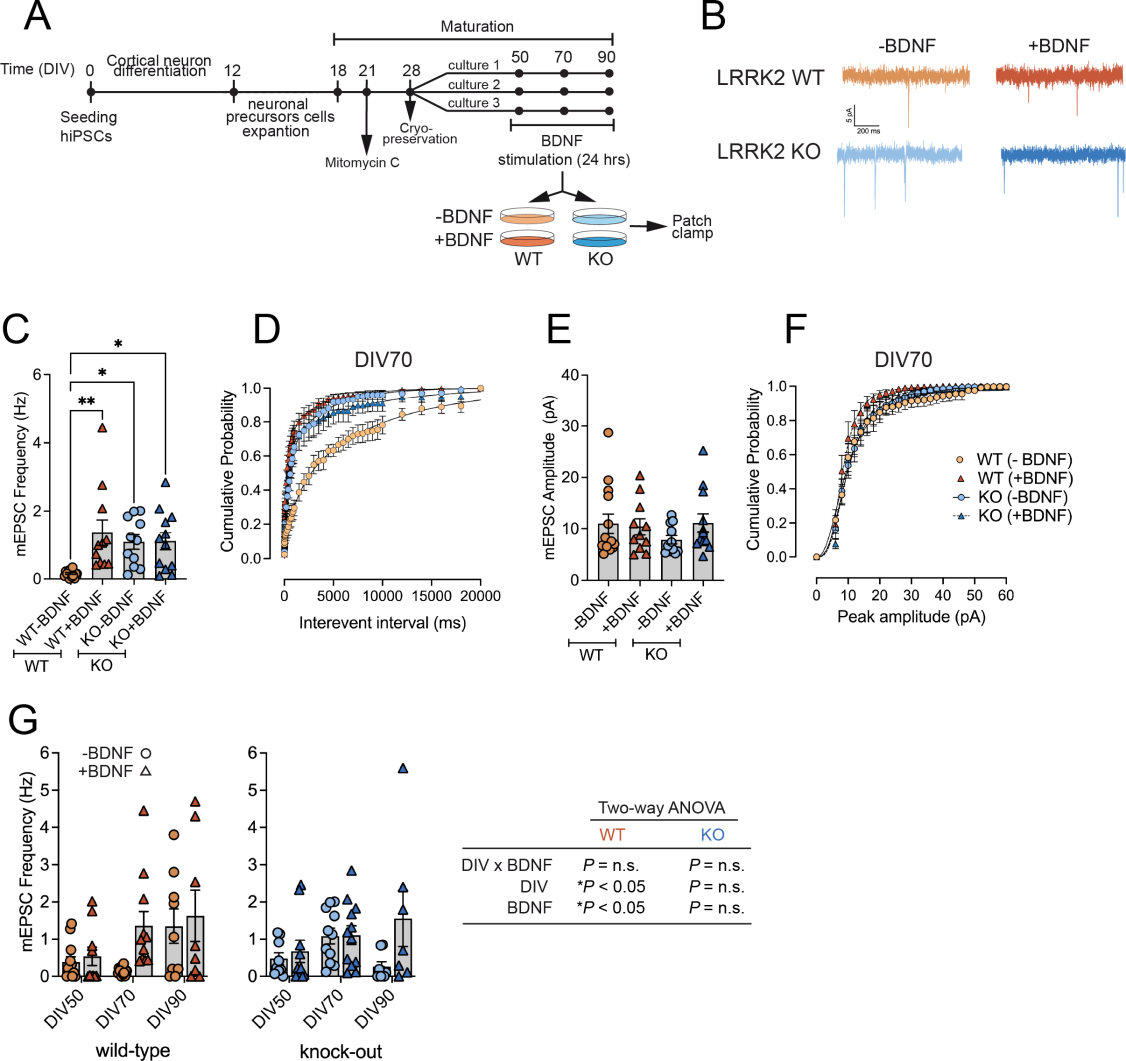
Effect of BDNF exposure on spontaneous electrical activity recorded in DIV70 LRRK2 WT and KO cortical neurons. (**A**) Schematic representation of the experimental setup. (**B**) Representative spontaneous traces of hiPSC-derived WT and KO neurons in the presence or absence of 50 ng/mL BDNF for 24 hr. (**C**) Frequency of miniature postsynaptic excitatory currents (mEPSC) in LRRK2 WT and KO cultures (WT vehicle: 0.16±0.03 Hz [n=15, N=3]; WT BDNF: 1.36±0.38 Hz [n=11, N=3]; KO vehicle: 1.09±0.21 Hz [n=11, N=3]; KO BDNF: 1.11±0.25 Hz [n=12, N=3]). Statistical significance was determined using one-way ANOVA (WT vehicle vs. WT BDNF **p<0.01; WT BDNF vs. KO BDNF *p<0.05; WT vehicle vs. KO BDNF *p<0.05; p>0.05 for all the other comparisons). (**D**) Cumulative probability curves of interevent interval (IEIs) in DIV70 WT and KO -/+ BDNF-treated cultures. (**E**) Amplitude of miniature postsynaptic excitatory currents (mEPSC) in DIV70 LRRK2 WT and KO cultures. BDNF treatment has no effect on the average peak amplitude (WT vehicle: 11.00±1.85 pA [n=14, N=3]; WT BDNF: 7.9±0.84 pA [n=11, N=3]; KO vehicle 10.42±1.53 pA [n=11, N=3]; KO BDNF 11.12±1.76 pA [n=12, N=3]). Statistical significance was determined using one-way ANOVA (p>0.05 for all comparisons). (**F**) Cumulative probability curves of peak interval in DIV70 WT and KO -/+ BDNF-treated cultures. (**G**) mEPSC frequency in WT and LRRK2 KO neurons at DIV50, DIV70 (data shown in C), and DIV90 under vehicle (circles) or BDNF (triangles) conditions. WT: DIV50 vehicle, 0.38±0.14 Hz (n=12, N=3); DIV50 BDNF, 0.54±0.25 Hz (n=10, N=3); DIV70 vehicle, 0.16±0.03 Hz (n=15, N=3); DIV70 BDNF, 1.36±0.38 Hz (n=11, N=3); DIV90 vehicle, 1.35±0.46 Hz (n=9, N=3); DIV90 BDNF, 1.63±0.69 Hz (n=8, N=3). KO: DIV50 vehicle, 0.48±0.16 Hz (n=9, N=3); DIV50 BDNF, 0.67±0.30 Hz (n=10, N=3); DIV70 vehicle, 1.09±0.21 Hz (n=11, N=3); DIV70 BDNF, 1.11±0.25 Hz (n=12, N=3); DIV90 vehicle, 0.26±0.13 Hz (n=8, N=3); DIV90 BDNF, 1.56±0.76 Hz (n=7, N=3). Two-way ANOVA (DIV × treatment) showed significant effects of DIV (F(2,59)=4.61, P=0.0138) and BDNF (F(1,59)=4.07, P=0.0482) in WT neurons, with no interaction (F(2,59)=1.69, P=0.1936); no significant effects were detected in KO neurons (DIV: F(2,51)=1.51, P=0.2299; BDNF: F(1,51)=3.70, P=0.0600; interaction: F(2,51)=2.10, P=0.1325). Bars represent mean ± SEM; n, cells; N, independent cultures.

To further explore the connection between LRRK2 and BDNF activity in a human disease-relevant model, we differentiated human induced pluripotent stem cells (hiPSCs) into cortical neurons following established protocols (Shi et al., 2012). Wild-type and isogenic LRRK2 knockout (KO) hiPSCs (Beylina et al., 2021) were differentiated in parallel for 50, 70, and 90 days (three independent culture rounds per genotype), prior to subjecting them to patch clamp to assess spontaneous activity in the absence or presence of acute exposure to BDNF (24 hr, 50 ng/mL; Figure 6A).

**Figure 6—figure supplement 1.**
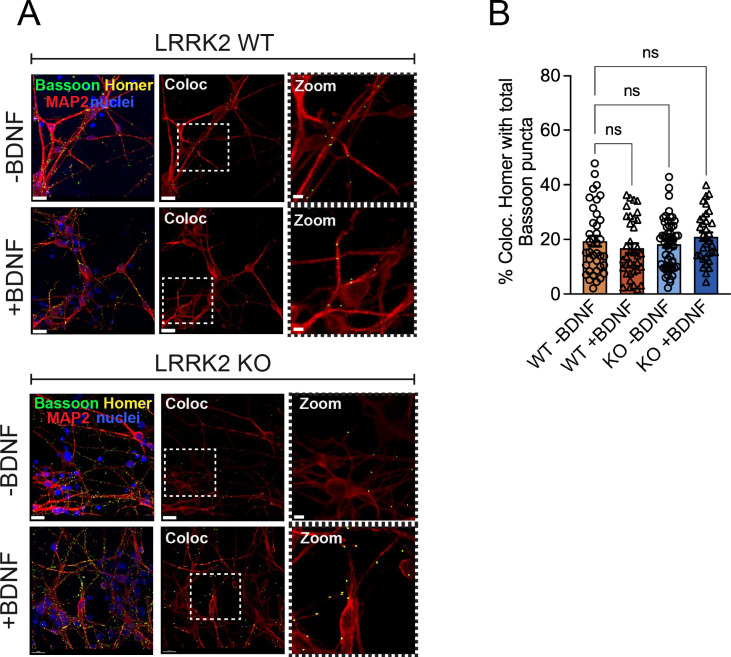
LRRK2 Deletion and BDNF Treatment Do Not Alter Bassoon–Homer Colocalization in Cortical Neurons. (**A**) Immunocytochemistry of WT and LRRK2 KO cortical neurons at DIV70 using Bassoon (pre-synaptic marker), Homer (post-synaptic marker), MAP2 (neuronal marker), and DAPI (scale bars 20 µm; zoom 5 µm). (**B**) Quantification of the % of Homer puncta colocalizing with total Bassoon (on MAP2-positive processes). BDNF was applied at 50 mg/mL for 24. One-way ANOVA with Tukey’s post-doc test. p>0.05 for all comparisons. N=4 cultures with 5–25 neurons imaged per condition per culture.

**Figure 6—figure supplement 2.**
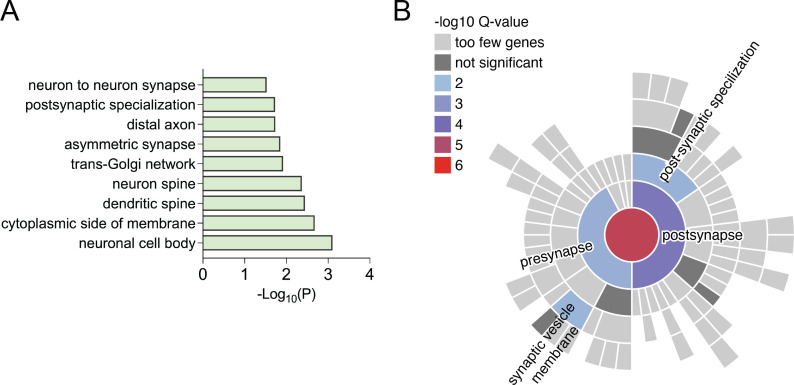
Neuronal and Synaptic Enrichment of Genes Associated with Significant GWAS Loci. (**A**) Functional enrichment analysis of the nearest genes to each of the 134 significant loci (obtained from table S3, column F from https://doi.org/10.1101/2025.03.14.24319455) was performed using g:Profiler g:GOSt with term size <500 to increase specificity. (**B**) SynGO analysis of 32 genes mapped from the input list (from GWAS) revealed significant enrichment of synaptic Cellular Component (5 terms) and Biological Process (6 terms) annotations at 1% FDR. Annotations were based on 31 genes for Cellular Components and 27 for Biological Processes, using a brain-expressed background of 18,035 genes. Experimental-evidence filtering was not applied. SynGO version 20231201 was used.

Acute BDNF treatment led to a significant increase in the mean frequency of miniature post-synaptic excitatory currents (mEPSC) in WT cultures (Figure 6B–C; Table 1). This effect was further supported by a leftward shift in cumulative probability curves of interevent intervals (IEIs) in BDNF-treated WT neurons compared with untreated controls (Figure 6D; Table 1). In contrast, LRRK2 KO neurons displayed elevated mEPSC frequency and did not show a significant response to BDNF treatment (Figure 6C–D, Table 1). Analysis of mEPSC peak amplitudes revealed no significant differences between genotypes or treatments, as reflected by comparable mean amplitudes and overlapping cumulative amplitude distributions (Figure 6E–F, Table 1), indicating that the observed effects primarily affect synaptic event frequency rather than postsynaptic receptor function. Consistently, two-way ANOVA performed across different developmental stages (DIV50, DIV70, DIV90) revealed significant main effects of DIV and BDNF treatment in WT neurons, whereas no significant effects of DIV, BDNF, or their interaction were detected in LRRK2 KO neurons (Figure 6G; Table 1). Together, these results indicate that synaptic activity in WT neurons is regulated by both neuronal maturation and BDNF signaling, whereas LRRK2 deficiency alters this regulation. The selective effect on mEPSC frequency, but not amplitude, at DIV70 suggests that BDNF predominantly modulates presynaptic function in WT neurons. Consistent with this interpretation, quantification of presynaptic Bassoon puncta colocalized with postsynaptic Homer revealed no increase in synapse number following BDNF treatment in either genotype (Figure 6—figure supplement 1C–D), supporting a model in which BDNF enhances neurotransmitter release probability rather than synapse formation.

**Table 1.** Capacitance and access resistance of LRRK2 KO and WT cortical neurons at 50, 70 and 90 DIV with or without BDNF stimulation.

Capacitance 50 DIV				Access resistance 50 DIV			
	Mean (pF)	SEM	n		Mean (MΩ)	SEM	n
WT - BDNF	14.14	1.858	12	WT - BDNF	18.54	1.312	12
WT +BDNF	15.1	1.331	12	WT +BDNF	18.27	1.053	12
KO - BDNF	16.21	2.299	9	KO - BDNF	20.11	1.467	9
KO +BDNF	14.66	1.295	11	KO +BDNF	18.71	1.884	11
**70 DIV**				**70 DIV**			
	Mean (pF)	SEM	n		Mean (MΩ)	SEM	n
WT - BDNF	23	2.177	16	WT - BDNF	21.18	1.372	16
WT +BDNF	22.4	2.579	12	WT +BDNF	21.6	1.273	12
KO - BDNF	24.64	2.555	13	KO - BDNF	22.36	1.835	13
KO +BDNF	20.03	2.232	13	KO +BDNF	15.1	1.122	13
**90 DIV**				**90 DIV**			
	Mean (pF)	SEM	n		Mean (MΩ)	SEM	n
WT - BDNF	12.51	1.994	8	WT - BDNF	17.6	2.221	8
WT +BDNF	20.9	6.365	7	WT +BDNF	18.64	1.68	7
KO - BDNF	24.23	3.062	7	KO - BDNF	16.66	1.609	9
KO +BDNF	24.96	6.49	8	KO +BDNF	14.64	1.591	8

## Discussion

Early synaptic alterations are thought to contribute to the pathogenesis of PD (Masotti et al., 2025), and understanding these early events is crucial to designing effective therapeutic approaches. Previous work from our laboratories and other groups has shown that the PD-associated kinase LRRK2 regulates important synaptic processes (Beccano-Kelly et al., 2014; Beccano-Kelly et al., 2015; Chen et al., 2025c). At the presynapse, LRRK2 controls the synaptic vesicle cycle through association with its WD40 domain (Maas et al., 2017; Cirnaru et al., 2014; Piccoli et al., 2014; Arranz et al., 2015) and phosphorylation of a panel of presynaptic proteins, including N-ethylmaleimide-sensitive fusion (NSF; Belluzzi et al., 2016), synapsin I (Marte et al., 2019), auxilin (Nguyen and Krainc, 2018), synaptojanin (Cao et al., 2017), and Endophylin A (Soukup et al., 2016). At the postsynapse, LRRK2 modulates the function of excitatory synapses in the striatum (Chen et al., 2020; Matikainen-Ankney et al., 2016; Tong et al., 2009; Volta et al., 2017; Beccano-Kelly et al., 2015; Xenias et al., 2022). However, the mechanisms by which LRRK2 affects synaptic function are still poorly elucidated. In the present study, we show that LRRK2 affects synaptic function in response to BDNF neurotrophic stimulation, promoting its relocalization to the actin-cytoskeleton in a phosphorylation-dependent manner. These findings support a model in which neurotrophic signaling engages LRRK2 to regulate cytoskeletal dynamics and synaptic function, shedding light into early synaptic dysfunction in PD.

Our study uncovers new insights into LRRK2 regulation by neurotrophic signaling, specifically identifying BDNF as an extracellular stimulus that transiently enhances LRRK2 activity. LRRK2 knockout neurons and differentiated SH-SY5Y LRRK2 KO cells show decreased activation of downstream AKT and ERK1/2, suggesting LRRK2 functions downstream of TrkB/BDNF and upstream of ERK1/2 and AKT. We observed high Ser935 phosphorylation under basal conditions, in agreement with mass spectrometry determination showing nearly complete phosphorylation of this residue in the whole brain (Nirujogi et al., 2021). However, RAB substrates and BDNF-responsive kinases like ERK and AKT remain minimally phosphorylated at baseline, indicating they stay responsive. BDNF exposure leads to reduced Ser935 phosphorylation post-stimulation, possibly due to negative feedback mechanisms, while RAB phosphorylation did not drop below pre-stimulation levels. These findings highlight the nuanced regulation of LRRK2 activity and indicate a positive correlation between Ser935 phosphorylation and LRRK2 activity under BDNF signaling, similar to previous observations on microglia cells upon inflammatory stimuli (Nazish et al., 2023).

Moreover, using a literature-based network analysis of LRRK2 PPIs, combined with AP-MS/MS in SH-SY5Y cells and phosphoproteomics of hyperactive LRRK2 G2019S from brain striatum, we identified a highly interconnected cluster of actin and synaptic proteins linked to LRRK2. Both BDNF-activated LRRK2 and hyperactive LRRK2 G2019S mutant are associated with GO terms related to synaptic function and cytoskeletal organization. The strong enrichment of synaptic components in the phosphoproteomic study is particularly notable given that the experiment was performed on total striatal lysates, which is a heterogeneous mixture of several cell types. This strengthens the importance of LRRK2-mediated signaling within the synaptic compartment.

The central role of synaptic dysfunction in PD neurodegeneration is increasingly recognized (Chen et al., 2025a). The latest and largest meta-analysis of PD genome-wide association studies (GWAS), which identified 134 loci as risk factors for PD further supports this notion (Leonard and Global Parkinson’s Genetics Program (GP2), 2025). The open-reading frames closest to these risk signals showed strong enrichment for neuronal genes involved in dendritic spine and synapse biology (Figure 6—figure supplement 2; Leonard and Global Parkinson’s Genetics Program (GP2), 2025). Even though these data require functional validation (GWAS identifies risk loci rather than causal genes), they provide a genetic rationale for synaptic dysfunction as a key contributor to PD pathophysiology (Uytterhoeven et al., 2025).

Given the strong connection of LRRK2 with PD (Paisán-Ruíz et al., 2004; Zimprich et al., 2004), elucidating the role of LRRK2 in synaptic biology will enhance our molecular understanding of how synaptic dysfunction contributes to PD (Masotti et al., 2025). Consistent with a synaptic role for LRRK2, we found that knockout of mouse *Lrrk2* resulted in blunted BDNF-induced synapse maturation in vitro. Moreover, BDNF-dependent enhancement of synaptic activity is detectable in WT human iPSC-derived cortical neurons but not in KO cells. Overall, these findings suggest a mechanism by which BDNF signaling reshapes the LRRK2 interactome to promote its incorporation into a macromolecular complex required for coordinating actin dynamics during synaptic structural plasticity.

BDNF plays a key role in reorganizing the actin cytoskeleton during long-term potentiation (LTP) via Rho GTPases Rac1 and Cdc42 (Hedrick et al., 2016). LRRK2 was previously reported to interact with Rac1 (Chan et al., 2011; Feng et al., 2018) and with other actin-related proteins (Meixner et al., 2011). Our study shows that LRRK2 associates with the actin cytoskeleton in dendritic spines, which increases upon BDNF stimulation. We also validated the BDNF-dependency of the interaction between LRRK2 and drebrin, an actin-binding protein that stabilizes F-actin in dendritic spines (Koganezawa et al., 2017). Evidence for a LRRK2-drebrin link includes increased phosphorylation at Ser339 in G2019S knockin striata and reduced drebrin levels in Lrrk2 knockout brains during development, correlating with morphological changes in dendritic protrusions. Ser339, found in the N-terminal region of human drebrin and which we identified as important for the interaction with LRRK2, lies just before the proline-rich (PP) domain of the protein. The N-terminal region of drebrin is located between the actin-binding domains and the C-terminal Homer-binding sequences, and it plays a key role in PPIs and cytoskeletal regulation (Worth et al., 2013). Notably, this region has been shown to interact with adafin, a protein involved in cytoskeletal-related processes crucial for synapse formation and function. Adafin regulates puncta adherentia junctions, presynaptic differentiation, and cadherin complex assembly, all of which are essential for hippocampal excitatory synapses, spine formation, and learning and memory processes (Beaudoin et al., 2012). Of note, adafin is listed among LRRK2-interacting proteins (https://www.ebi.ac.uk/intact/home), supporting a potential functional connection between LRRK2-mediated drebrin phosphorylation and the formation of adafin-drebrin complex.

It has been shown that drebrin knockout results in delayed synaptogenesis and inhibition of postsynaptic PSD95 accumulation, indicating its key role in spine maturation (Koganezawa et al., 2017; Takahashi et al., 2003; Aoki et al., 2009). During LTP, Ca^2+^ entry through N-methyl-d-aspartate receptors causes drebrin exodus from the spine compartment. This allows actin monomers to enter and polymerize, ultimately increasing the size of the cytoskeleton (Sekino et al., 2006). We postulate that LRRK2 may promote drebrin exodus at the shaft of the dendrite during plasticity processes, consistent with our previous work showing that LRRK2 regulates a PKA translocation from the shaft to dendritic spines (Cho et al., 2014). Actin remodeling during spine enlargement is also coordinated by the ARP2/3 complex, which is activated by binding to WAVE family proteins downstream of active Rac1 (Spence et al., 2016). Strikingly, we found that BDNF stimulates LRRK2 interaction with three out of seven subunits of the ARP2/3 complex (ACTR2, ACTR3, and ARPC2), and LRRK2 was reported to interact with WAVE proteins (Kim et al., 2018). Altogether these findings, in agreement with our earlier study (Parisiadou et al., 2014), highlight a crucial role for LRRK2 in actin-based dendritic spine remodeling. Clearly, future studies should focus on clarifying the precise molecular dynamics and kinetics of these processes.

Actin dynamics underlie key developmental processes. In this context, our study uncovers a developmental phenotype in which loss of Lrrk2 leads to defects in dendritic protrusions morphogenesis. Interestingly, this developmental alteration is rescued with age. This is not surprising considering that the homologous kinase LRRK1 may compensate for LRRK2 deficiency, as suggested by the overt neurodegeneration observed in double *Lrrk1/Lrrk2* KO mice but absent in the single KO (Giaime et al., 2017; Huang et al., 2022; Kang et al., 2024) and the redundant but not overlapping pattern of expression of the two kinases (Biskup et al., 2007). Indeed, striatal Lrrk2 expression in the rodent brain increases up to postnatal week 4 (Parisiadou et al., 2014; Westerlund et al., 2008; Biskup et al., 2007; Cogo et al., 2022), while the Lrrk1 transcript in this region remains relatively stable during development (Westerlund et al., 2008).

Several limitations of this study should be acknowledged. First, biochemical analyses based on western blotting revealed variability in protein abundance and phosphorylation levels across experiments, likely reflecting both biological heterogeneity and technical constraints inherent to bulk tissue and cell-based assays. Second, although our electrophysiological analyses revealed genotype and BDNF-dependent effects, these measurements were performed on a limited number of independent cultures and focused on cortical neurons. Future studies using larger datasets and additional neuronal cell types will be necessary to validate and extend these findings. In particular, the study of dopaminergic neurons, the population most vulnerable in PD, is deemed critically important, especially considering the recent findings highlighting subtype-specific vulnerability of dopaminergic neurons in PD to account for high heterogeneity even within dopamine neurons (Masotti et al., 2025; Chen et al., 2025c; Gaertner et al., 2025).

Several lines of evidence indicate that genetic, biochemical, and cellular alterations of BDNF/TrkB signaling are linked to PD. At striatal synapses, BDNF/TrkB signaling enhances both dopamine and glutamate release and ERK1/2 activation (Narita et al., 2003; Morella et al., 2020). BDNF is required for the establishment of the proper number and for the survival of dopaminergic neurons in the SNpc (Hyman et al., 1991; Baquet et al., 2005), and presynaptic dopamine release in the striatum is enhanced by BDNF (Baydyuk and Xu, 2014). BDNF levels are lower in peripheral tissues, brain, and blood of sporadic PD patients (Rahmani et al., 2019). Moreover, the V66M polymorphism modifies the risk of sporadic and mutant LRRK2-associated PD (Lin et al., 2011; Liu et al., 2012). Decreased BDNF concentration in serum and brain correlates with an increased degeneration of dopaminergic neurons in PD (Siuda et al., 2017) and with loss of striatal DA transporter in patients with striatal dopaminergic neurodegeneration (Ziebell et al., 2012). Future studies should investigate whether BDNF signaling is altered in G2019S mice and hiPSCs-derived neurons with the G2019S mutation.

At the onset of PD, loss of dopaminergic axonal terminals exceeds the loss of cell bodies (Soukup et al., 2018), implying that early synapse deterioration may trigger axonal degeneration and, ultimately, neuronal death (Tagliaferro and Burke, 2016). In contrast to neuronal loss, which is irreversible, disease-associated synaptic dysfunctions could be rescued through the formation of new axons (Picconi et al., 2012). Thus, focusing on early, prodromal dysfunction in PD appears key to designing effective therapeutic and preventive strategies, and our study uncovered LRRK2 as a promising disease-modifying target of early-stage PD.

## Materials and methods

**Key resources table:** 

Reagent type (species) or resource	Designation	Source or reference	Identifiers	Additional information
Genetic reagent (*Mus musculus*)	*Lrrk2* knock-out (KO) mice	Mayo Clinics	JAX:016121; RRID:IMSR_JAX:016121	Provided by Dr. Heather Melrose
Genetic reagent (*M. musculus*)	G2019S-*Lrrk2* knock-in (KI) mice	Novartis	MGI:5295368; registered Novartis *Lrrk2tm1.1Npa* allele	Provided by Prof. Michele Morari and Dr. Derya R Shimshek
Genetic reagent (*M. musculus*)	*Dbn1* knock-out (KO) mice	Previously described	MGI:5501061; PMID:28198431; the relevant allele is *Dbn1tm1b(KOMP)Wtsi*.	See Willmes et al., 2017
Genetic reagent (*Homo sapiens*)	LRRK2 KO SH-SY5Y monoclonal cell line	Generated in this study	Not available	Generated by CRISPR/Cas9
Genetic reagent (*H. sapiens*)	GFP-LRRK2 wild type expressing SH-SY5Y cells	Generated in this study	Not available	
Biological sample (*M. musculus*)	Primary cortical neurons	This study	Not available	Isolated from P0 C57BL/6 J mice and *Lrrk2* knock-out (KO) mice
Cell line (*H. sapiens*)	SH-SY5Y human neuroblastoma cell line	Interlab Cell Line Collection (ICLC)	HTL95013, RRID:CVCL_0019	Originally from ECACC; authenticated by STR profiling; mycoplasma negative
Cell line (*H. sapiens*)	HEK293T	ATCC	ATCC CRL-3216; RRID:CVCL_0063	Human embryonic kidney cell line
Cell line (*H. sapiens*)	Isogenic WT and LRRK2 KO hiPSC lines	Mark R. Cookson laboratory	RRID:CVCL_RM92; RRID:CVCL_A8ZU; RRID:CVCL_A8ZV	Provided by Mark R. Cookson’s laboratory
Antibody	α-LRRK2 (rabbit monoclonal)	Abcam	Cat#: ab133474 RRID:AB_2713963	WB (1:300)
Antibody	α-Phospho-Ser935 (rabbit monoclonal)	Abcam	Cat# ab133450, RRID:AB_2732035	WB (1:300)
Antibody	α–21Phospho-Ser473-AKT (mouse monoclonal)	Santa Cruz Biotechnology	Cat# sc-293125, RRID:AB_2847909	WB (1:500)
Antibody	α-AKT (rabbit polyclonal)	Cell Signaling Technology	Cat# 9272, RRID:AB_329827	WB (1:1000)
Antibody	α-p44/42 MAPK (Erk1/2) (rabbit monoclonal)	Cell Signaling Technology	Cat# 4695, RRID:AB_390779	WB (1:1000)
Antibody	Α-phospho Erk1/2 (Thr202 /Tyr204, Thr185/Tyr187) (rabbit monoclonal)	Millipore	Cat# 05–797 R RRID:AB_1587016	WB (1:1500)
Antibody	α-βIII tubulin (mouse monoclonal)	Sigma-Aldrich	Cat# T8578, RRID:AB_1841228	WB (1:40,000)
Antibody	α-Drebrin (mouse monoclonal)	Thermo Fisher Scientific	Cat# MA1-20377, RRID:AB_2089494	WB (1:500) IF (1:400)
Antibody	α-Flag M2-HRP (mouse monoclonal)	Sigma-Aldrich	Cat# A8592, RRID:AB_439702	WB (1:5000)
Antibody	α-Mouse IgG-HRP (rabbit polyclonal)	Sigma-Aldrich	Cat# A9044, RRID:AB_258431	WB (1:80,000)
Antibody	α-Rabbit IgG-HRP (goat polyclonal)	Sigma-Aldrich	Cat# A9169, RRID:AB_258434	WB (1:16,000)
Antibody	α-PSD95 (mouse monoclonal)	Abcam	Cat# ab2723, RRID:AB_303248	IF (1:200)
Antibody	α-MAP2 (rabbit polyclonal)	Santa Cruz Biotechnology	Cat# sc-20172, RRID:AB_2250101	IF (1:200)
Antibody	α-MAP2 (chicken polyclonal)	Antibodies	Cat# A85363, RRID:AB_2748940	IF (1:1000)
Antibody	α-Homer 1 (rabbit polyclonal)	Synaptic Systems	Cat# 160 003, RRID:AB_887730	IF (1:500)
Antibody	α-Bassoon (guinea pig polyclonal)	Synaptic Systems	Cat# 141 004, RRID:AB_2290619	IF (1:500)
Antibody	α-Mouse-Alexa Fluor 568 (goat polyclonal)	Molecular Probes	Cat# A-11004, RRID:AB_2534072	IF (1:200)
Antibody	α-Rabbit-Alexa Fluor 488 (goat polyclonal)	Molecular Probes	Cat# A-11034, RRID:AB_2576217	IF (1:200)
Antibody	α-Rabbit-Alexa Fluor 568 (goat polyclonal)	Molecular Probes	Cat# A-11036, RRID:AB_10563566	IF (1:200)
Antibody	α-Rabbit-Alexa Fluor 405 (goat polyclonal)	Molecular Probes	Cat# A-31556, RRID:AB_221605	IF (1:200)
Antibody	α-Guinea pig-Alexa-488 (goat polyclonal)	Molecular Probes	Cat# A-11073, RRID:AB_2534117	IF (1:200)
Antibody	α-Chicken-Alexa 647 (goat polyclonal)	Molecular Probes	Cat# A-21449, RRID:AB_2535866	IF (1:200)

### Mouse strains

C57BL/6 J *Lrrk2* knock-out (KO) were provided by Dr. Heather Melrose (Mayo Clinics, Florida, USA; JAX:016121). G2019S-Lrrk2 knock-in mice were obtained from Prof. Michele Morari and Novartis Institutes for BioMedical Research, Novartis Pharma AG (Basel, Switzerland). C57BL/6 J or *Dbn1* KO were previously described (Willmes et al., 2017). Housing and handling of mice were done in compliance with national guidelines. All animal procedures were approved by the Ethical Committee of the University of Padova and the Italian Ministry of Health (license 1041/2016-PR and 105/2019), by the ‘Landesamt für Gesundheit und Soziales’ (LaGeSo; Regional Office for Health and Social Affairs in Berlin) under the permit number T-CH0025/23, and followed the guidelines approved by the Northwestern University Animal Care and Use Committee. Approximately equal numbers of males and females were used for every experiment.

### Cell cultures

#### Generation of LRRK2 KO SH-SY5Y CRISPR/Cas9 edited monoclonal cell line

The KO of LRRK2 was performed using CRISPR/Cas9-mediated genome editing technology following the protocol by Sharma et al., 2018. Two sgRNAs were selected among those designed by the laboratory of Zhang F (here) and one was designed using the online platform Benchling (https://www.benchling.com). All the gRNAs were synthesized by Sigma-Aldrich. The oligos pairs encoding the 20-nt guide sequence were annealed and ligated into the pSpCas9(BB)–2A-Puro (PX459) V2.0 vector (Addgene, Watertown, MA, US) and then amplified in chemically competent *Escherichia coli* StbI3 cells (Thermo Fisher Scientific). Human neuroblastoma-derived SH-SY5Y cells were transfected using Lipofectamine 2000 (Invitrogen) and subjected to puromycin selection. The selected cells were diluted to obtain monoclonal cell lines. Approximately 1 week after plating, the colonies were inspected for a clonal appearance and progressively expanded. Finally, the deletion of LRRK2 was verified in multiple lines by western blot analysis with LRRK2-specific antibody (Figure 4—figure supplement 1).

#### SH-SY5Y cell line maintenance, differentiation and treatments

The SH-SY5Y cell line (ICLC HTL95013) was obtained from the Interlab Cell Line Collection (ICLC; originally sourced from ECACC). Cell line identity was authenticated by STR profiling and further validated by isoenzyme analysis. Cells were confirmed to be free of mycoplasma contamination by Hoechst staining and PCR.

SH-SY5Y cells (naive, LRRK2 KO, expressing GFP, or GFP-LRRK2 wild type) were cultured in a mixture (1:1) of Dulbecco’s Modified Eagle’s Medium (DMEM, Biowest) and Ham’s F-12 Nutrient Mixture (F12, Biowest), supplemented with 10% (v/v) FBS (Thermo Fisher Scientific) and 1% Penicillin/Streptomycin solution (PS, GIBCO Life Technologies).

To promote N-type (neuronal-like cells) cell differentiation, cells were plated in DMEM/F12 containing 1% PS, 1% FBS, and 10 μM of all-trans-RA (Sigma-Aldrich). At regular intervals of 48 hrs, RA was newly provided to the medium. Cells were differentiated for 6 days and then subjected to the treatments.

SH-SY5Y cells were starved for 5 hr in DMEM/F12 supplemented with 1% PS and then stimulated with 100 ng/mL BDNF (50240-MNAS, Sino Biological) for different time periods (5, 15, 30, and 60 min) in serum-free medium. LRRK2 kinase activity was inhibited pre-treating cells with 0.5 μM MLi-2 (ab254528, Abcam) for 90 min. For the detection of phospho-S935 and phospho-Rabs, 80  µg of total protein were loaded, whereas 30  µg were used for the detection of phospho-AKT and phospho-ERK1/2, as well as for total proteins.

#### HEK293T cell line maintenance and transfection

The HEK293T cell line was obtained from the American Type Culture Collection (ATCC). Cell line identity was authenticated by STR profiling. Cells were confirmed to be free of mycoplasma contamination by Hoechst staining and PCR.

HEK293T cells were cultured in DMEM (Biowest) supplemented with 10% (v/v) FBS (Thermo Fisher Scientific) and 1% PS (GIBCO Life Technologies). Cells were plated in 100 mm dishes and, once they reached 80% confluency, they were transfected with polyethyleneimine (PEI, Polysciences) at a 1:2 DNA to PEI ratio (v/w). Flag-LRRK2 was co-expressed with YFP-drebrin full-length, N-terminal domain (1–256 aa), or C-terminal domain (256–649 aa).

#### Primary mouse cortical neuron preparation, transfection, and treatments

Primary cortical neurons from *Lrrk2* WT and KO C57BL/6 J mice were derived from postnatal mouse (P0) exploiting the Papain Dissociation System (Worthington Biochemical Corporation). Cortices were incubated in Papain solution (Papain and DNase solution in Earle’s Balanced Salt Solution) for 40 min at 37 °C. Subsequently, tissue was triturated and centrifuged for 5 min at 200 × *g*. The supernatant was discarded, and the pellet was resuspended in Stop solution [DNase solution and Trypsin inhibitor solution (15.5 mg/mL) in Earle’s Balanced Salt Solution]. For 10 min, the tissue was allowed to precipitate and then the supernatant was pipetted drop-by-drop on 5 mL of 10/10 solution (10 µg/mL Trypsin inhibitor and 10 µg/mL BSA in Earle’s Balanced Salt Solution) and centrifuged for 10 min at 100 × *g*. The pellet was resuspended in Neurobasal A medium (GIBCO Life Technologies) supplemented with 2% B27 supplement (GIBCO Life Technologies), 0.5 mM L-glutamine (GIBCO Life Technologies), 100 units/mL penicillin, and 100 μg/mL streptomycin (GIBCO Life Technologies). Cells were diluted and counted in 0.4% Trypan blue. Neurons were plated at 1000–1500 cells/mm^2^ onto 6-well plates or at 200 cells/mm^2^ on 12 mm glass coverslips in 24-well plates and maintained in culture for 14 days prior to the treatments. After 7 days, 50% of the Neurobasal medium was removed and replaced with a fresh one.

At DIV14, *Lrrk2* WT and KO mature neurons cultured in the six-well plates for western blot analysis were treated with MLi-2 (0.5 μM) for 90 min and with BDNF (100 ng/mL) for 5, 30, 60, and 180 min in Neurobasal completed medium. At DIV4, Lrrk2 WT and KO primary neurons plated in 24-well plates were transfected with lipofectamine (Lipofectamine 2000, Invitrogen) in a 1:2 ratio with DNA (v/w). The transfection was carried out in Opti-MEM (GIBCO Life Technologies) for 45 min. Transfected neurons were then maintained in culture for an additional 9–10 days. At DIV14, neurons were treated for 24 hr with 100 ng/mL BDNF to study the spine maturation process.

#### Media composition for cortical neuron differentiation from hiPSC

Neuronal maintenance media (NMM): 1:1 mixture of N2 media DMEM/F12 GlutaMax supplemented with 1X N2, 50 units/mg/mL Penicillin/Streptomycin solution, 1 X MEM Non-Essential Amino Acids Solution [100 X], 100 µM 2-Mercaptoethanol [ThermoFisher Scientific], 1 mM sodium pyruvate, and 5 µg/mL insulin [Merck] and B27 media Neurobasal supplemented with 1 X B27 and 2 mM L-Glutamine (ThermoFisher Scientific).

Neuronal induction media (NIM): NMM media supplemented with 100 nM LDN193189 (Sigma Aldrich) and 10 µM SB431542 (Bio-Techne).

#### hiPSC cell culture, cortical neuron differentiation, and treatments

Isogenic human induced-pluripotent stem cell lines (hiPSC) of WT and LRRK2 KO, made and kindly provided by Mark R. Cookson’s laboratory (Beylina et al., 2021) were cultured using E8 media (Thermo Fisher Scientific) in a six-well plate coated with Cultrex. Cortical neuronal differentiation was carried out according to Shi et al., 2012. Briefly, upon reaching 100% confluency, media was changed to neuronal induction media (NIM) and is marked as 0 days in vitro (DIV0). Daily NIM media change was carried out till DIV12, upon which neuronal precursor cells (NPCs) are generated. On DIV12, NPCs were expanded via passage at a 1:2 ratio, supplemented with neuronal maintenance media (NMM) media and 20 ng/mL FGF2 (Bio-Techne). Neurogenesis was initiated on DIV18 with the removal of FGF2 and the cells supplemented with NMM only changing every 2 days. At DIV25-30, neurons were cryopreserved in CryoStor CS10 solution (Stem Cell Technologies).

For electrophysiological recordings and immunocytochemistry/immunofluorescence (ICC/IF), thawed neurons were plated on Cultrex coated 10 mm coverslips at a density of 60 k/cm^2^. All cells were treated with 1 ug/mL Mitomycin C (Sigma-Aldrich) for 1 hr at 37 °C, 48–72 hr after plating to remove any proliferating cells. Neurons were maintained in NMM until used for experiments. BDNF treatment was carried out with cells incubated with 50 ng/mL BDNF (Bio-Techne) for 24 hr prior to use in electrophysiological recordings and ICC/IF.

#### Electrophysiological recordings

Whole-cell patch-clamp recordings were performed on cortical neurons at DIV70-75 (mentioned as DIV70 from here on) in voltage clamp at Vh −70 mV and the membrane test function was used to determine intrinsic membrane properties ∼1 min after obtaining whole-cell configuration, as described previously (Beccano-Kelly et al., 2023). Briefly, coverslip containing neurons were transferred to Warner bath (RC-26G) and maintained at 35 ± 2 °C through constant perfusion using in-line heater and controller (Warner Instruments) with extra cellular solution (ECS) containing (in mM unless stated): 145 NaCl, 3 KCl, 2 MgCl_2_, 2 CaCl_2_, 10 glucose, 10 HEPES, pH 7.4, 310 mOsm. Tetrodotoxin (TTX 0.2 μM) and picrotoxin (PTX 100 μM) were added before use to block sodium and GABA_A_ currents. Pipette resistance (Rp) of glass electrodes was 4–6 MΩ when filled with (in mM): 130 Cs-methanesulfonate, 5 CsCl, 4 NaCl, 2 MgCl2, 5 EGTA, 10 HEPES, 5 QX-314, 0.5 GTP, 10 Na2-phosphocreatine, and 5 MgATP, 0.1 spermine, pH 7.2, 300 mOsm. Data was acquired by Multiclamp700B amplifier and Digidata 1550B and signals were filtered at 2 kHz, digitized at 10 kHz, and analyzed in Clampfit 10 (Molecular Devices). Tolerance for series resistance (Rs) was <35 MΩ and uncompensated; ΔRs tolerance cut-off was <20%. mEPSCs were analyzed using Clampfit 10 (threshold 5 pA); all events were checked by eye. Data are presented as mean ± SEM where n is cells from a minimum of 3 separate cultures (culture N in brackets).

#### Cells and tissues lysis, SDS-PAGE, and western blotting analysis

Immortalized cells, neurons, and mouse brain tissues were lysed for 30 min on ice in an appropriate volume of cold RIPA lysis buffer (Cell Signaling Technologies) supplemented with protease inhibitor cocktail. Protein concentration was assessed by performing BCA assay (Thermo Fisher Scientific). Proteins were solubilized in sample buffer (200 mM Tris-HCl pH 6.8, 8% SDS, 400 mM DTT, 40% glycerol, q.s. Bromophenol Blue) and resolved by SDS-PAGE on 8% Tris polyacrylamide homemade gels in Tris-glycine-SDS running buffer for the AP-MS experiments, and on 4–20% polyacrylamide gels (GenScript Express Plus PAGE) in Tris-MOPS-SDS running buffer (GenScript Running Buffer Powder) for the other cases. Proteins were transferred on PVDF (polyvinylidene difluoride, Bio-Rad) membranes using a semi-dry transfer system (Trans-Blot Turbo Transfer System, Bio-Rad). Non-specific binding sites were blocked by incubating membranes with 5% non-fat dry milk diluted in 0.1% Tween-20 Tris-buffered saline (TBS-T) for 1 hr at room temperature under agitation. Membranes were subsequently incubated overnight at 4 °C with primary antibodies in 5% non-fat dry milk in TBS-T or in 5% BSA in TBS-T. After three washes in TBS-T at room temperature, membranes were incubated for 1 hr at room temperature with horseradish peroxidase (HRP)-conjugated goat anti-mouse or anti-rabbit IgG. After three more washes in TBS-T, proteins were visualized using chemiluminescence (Immobilon ECL western HRP substrate, Millipore). Densitometric analysis was carried out using the Image J software. The antibodies used for western blotting are as follows: rabbit α-LRRK2 (MJFF2 c41-2, ab133474, Abcam, 1:300); rabbit α-phospho-Ser935 LRRK2 (ab133450, Abcam, 1:300); mouse α-phospho-Ser473-AKT (sc-293125, Santa Cruz Biotechnology, 1:500); rabbit α-AKT (9272 S, Cell Signaling Technology, 1:1000); rabbit α-phospho (Thr202/Tyr204, Thr185/Tyr187)-ERK1/2 (12–302, Millipore, 1:1500); rabbit α-ERK1/2 (4695, Cell Signaling Technology, 1:1000); mouse α-GAPDH (CSB-MA000195, 1:5000); mouse α-βIII tubulin (T8578, Sigma-Aldrich, 1:40000); mouse α-DREBRIN (MA1-20377, Thermo Fisher Scientific, 1:500); α-Flag M2-HRP (A8592, Sigma-Aldrich, 1:5000); α-mouse IgG-HRP (A9044, Sigma-Aldrich, 1:80000); α-rabbit IgG-HRP (A9169, Sigma-Aldrich, 1:16000).

### Staining on mammalian cells and brain tissue

#### Immunocytochemistry and confocal microscopy of primary neurons

Mouse primary cortical neurons derived from *Lrrk2* WT C57BL/6 J and KO pups (P0) were fixed using 4% paraformaldehyde (PFA, Sigma-Aldrich) in PBS1X pH 7.4 for 20 min at room temperature and, after three washes in PBS1X, they were subjected to staining protocol. Cells were firstly permeabilized with 0.3% Triton-X in PBS1X for 5 min and then saturated in blocking buffer (1% BSA Fraction V, 0.1% Triton-X, 50 mM Glycine, 2% goat serum in PBS1X) for 1 hr at room temperature in agitation. The primary and secondary antibodies incubation steps were carried out in working solution (20% blocking buffer in PBS1X) for 1 hr at room temperature. Both incubations were followed by three washes in working solution. Nuclei staining was performed in Hoechst 33258 (Invitrogen, 1:10,000 dilution in PBS1X) for 5 min and followed by three rinses in PBS1X. After being cleaned in distilled water, coverslips were mounted on microscope slides with Mowiol mounting medium. Immunofluorescence z-stack images (z-stack thickness: 0.5 μm × 6=3 μm) were obtained on a Zeiss LSM700 laser scanning confocal microscope exploiting a ×63 oil immersion objective.

The antibodies used for immunocytochemistry are as follows: mouse α-PSD95 (ab2723, Abcam, 1:200), rabbit α-MAP2 (sc-20172, Santa Cruz, 1:200); mouse α-drebrin (MA1-20377, Thermo Fisher Scientific, 1:400); goat α-mouse-Alexa Fluor 568 (A11004, Molecular Probes), goat α-rabbit-Alexa Fluor 488 (A11034, Molecular Probes), goat α-rabbit-Alexa Fluor 568 (A11036, Molecular Probes), and goat α-rabbit-Alexa Fluor 405 (A31556, Molecular Probes).

#### Immunostaining and image analysis of hiPCS-derived cortical neurons

For immunocytochemistry, DIV70 cells on coverslips were fixed in 4% paraformaldehyde (PFA) for 10 min. Cells were then permeabilized and blocked using 0.4% Tween with 10% normal goat serum (NGS) in PBS1X for 1 hr. Primary antibodies were incubated overnight at 4 °C in PBST plus 10% NGS. Cells were washed 3× with PBST before 1 hr incubation at room temperature with secondary antibodies (α-guinea pig Alexa-488, α-rabbit Alexa 568, α-chicken 647 along with DAPI) from Molecular Probes and Jackson Laboratories. Primary antibodies were chicken anti-microtubule associated protein 2 (MAP2) (Antibodies A85363, dil. 1:1000), rabbit anti-homer 1 (Synaptic Systems 160003, dil. 1:500), and guinea pig anti-bassoon (Synaptic Systems 141004, dil. 1:500). Coverslips were slide mounted with FluorSave (Sigma-Aldrich), and all images were acquired on a Leica confocal microscope as 0.45 μm z-stacks at ×60 magnification (flattened with the max projection function for cluster analysis). Single ×60 images were used for analyses. Excitation and acquisition parameters were constrained across all experimental paired (culture) acquisitions. All images were analyzed using IMARIS microscopy image analysis software (v10.0.0 Oxford Instruments). Data are presented as mean ± SEM.

#### Golgi-Cox staining

Animals were terminally anesthetized and transcardially perfused with 0.9% saline. Half of each brain was incubated with Golgi-Cox solution (Potassium dichromate, Mercuric chloride, Potassium chromate) prepared according to Zaqout and Kaindl, 2016 in the dark at room temperature for 14 days and then transferred in 30% sucrose solution in PBS1X. Brains were cut with a vibratome in 100-μm-thick slices. The sections were then blotted by pressing an absorbent paper moistened with sucrose solution onto the slides and dried for 7–10 min. The samples were then subjected to a color development procedure consisting of the following steps: (1) two 5 min washes in distilled water; (2) 5 min dehydration step in 50% ethanol; (3) 10 min incubation step in 20% ammonium hydroxide; (4) 5 min wash in distilled H_2_O; (5) 8-min incubation step in 5% sodium thiosulfate at room temperature in the dark; (6) two additional 1 min rinses in distilled H_2_O; (7) dehydration in ascending grades of ethanol (70%, 95%, and 100%). After two final 6 min incubations with xylene, the slides were covered with Eukitt mounting medium. Z-stack images (z-stack thickness between 0.5 μm × 30=15 μm and 0.5 μm × 80=40 μm) were acquired with a Zeiss LSM700 laser scanning confocal microscope using a 100 X/1.40 Oil DIC M27 immersion objective with phase contrast acquisition mode. All images were analyzed using freely available RECONSTRUCT software and according to Risher et al., 2014, three to four neurons per mouse (n=3 per genotype) were acquired in the dorsal striatum and n≌4 segments per neuron were analyzed.

#### Electron microscopy

Coronal brain slices were fixed with 2.5% glutaraldehyde in 0.1 M sodium cacodylate pH 7.4 buffer overnight at 4 ° C, postfixed in 1% osmium tetroxide for 1 hr at 4 ° C, ethanol dehydrated, then infiltrated in a mixture of EMbed 812 (Electron Microscopy Sciences) epoxy resin and absolute ethanol 1:1, and then finally embedded in EMbed 812 (Electron Microscopy Sciences) epoxy resin. Ultrathin sections were obtained with a Leica EM UC7 ultramicrotome and subsequently counterstained with uranyl acetate and lead citrate. Samples were then examined with a Tecnai G2 (Fei-Thermo Fisher) transmission electron microscope operating at 120 kV, digital images were acquired using a Veleta (Olympus Soft Imaging Solutions) digital camera. Images were acquired at the level of dorsal striatum in n=4 mice per genotype (1-month-old mice) or n=3 mice per genotype (18-months-old mice). n≌20 synapses per mouse were analyzed in 1-month-old mice; n≌40 synapses per mouse were analyzed in 18-months-old mice. We gratefully thank the DeBio Imaging facility at the University of Padova for their support with the staining procedure.

#### Quantitative PCR

Total RNA was isolated from midbrain, striatum, and cortex of 1-month-old animals (n=6 animals per group, Lrrk2 WT and Lrrk2 KO) using Animal Tissue RNA Purification Kit (Norgen), according to the manufacturer’s instructions. After extraction, RNA concentration was quantified with NanoDrop 2000C spectrophotometer (Thermo Fisher Scientific). After DNAse treatment (Thermo Fisher Scientific, according to the manufacturer’s protocol), complementary DNA (cDNA) was generated using qRT SuperMix (Bimake). The cDNAs were used for quantitative PCR (qPCR) exploiting iTaq Universal SYBR Green Supermix and CFX96 RealTime System (Bio-Rad) for 40 cycles. All samples were run in triplicate, and transcript levels normalized against the geometrical means of RPL27, actin, and GAPDH relative abundance (housekeeping genes). Data shown were produced using Bio-Rad CFX Manager software and analyzed according to ddCt algorithm.

Primers (5’–3’) used are:

*Bdnf* FW: GGCTGACACTTTTGAGCACGTC
*Bdnf* REV: CTCCAAAGGCACTTGACTGCTG
*Nrtk2* FW: TGAGGAGGACACAGGATGTTGA
*Nrtk2* REV: TTCCAGTGCAAGCCAGTATCTG
*Shank3* FW: ACCTTGAGTCTGTAGATGTGGAAG
*Shank3* REV: GCTTGTGTCCAACCTTCACGAC
*Psd95* FW: GGTGACGACCCATCCATCTTTATC
*Psd95* REV: CGGACATCCACTTCATTGACAAAC
*Arpc2* FW: GAGTCACAGTAGTCTTCAGCACG
*Arpc2* REV: AGGTTCCCTGTGGCTGAAAAGG
*Dbn1* FW: AGAAGTCGGAGTCAGAGGTGGA
*Dbn1* REV: ATGCCACTCGTTCCTGCTGTCT
*Darpp32* FW: AGATTCAGTTCTCTGTGCCCG
*Darpp32* REV: GGTTCTCTGATGTGGAGAGGC housekeeping genes
*Rpl27* FW: AAGCCGTCATCGTGAAGAACA
*Rpl27* REV: CTTGATCTTGGATCGCTTGGC
*Gapdh* FW: AGGTCGGTGTGAACGGAT TTG
*Gapdh* REV: TGTAGACCATGTAGTTGAGGTCA
*Actb* FW: CAACGGCTCCGGCATGTG
*Actb* REV: CTCTTGCTCTGGGCCTCG

#### Protein purification from mammalian cells

For the affinity purification (AP) protocol, SH-SY5Y GFP and SH-SY5Y GFP-LRRK2 cells were plated onto 100 mm dishes and differentiated for 6 days. SH-SY5Y GFP-LRRK2 cells were treated with BDNF (100 ng/mL) or with an equal volume of vehicle for 15 min, while SH-SY5Y OE-GFP cells were left untreated. Cells were harvested in lysis buffer (20 mM Tris-HCl pH 7.5, 150 mM NaCl, 1 mM EDTA, 1% Tween 20, 2.5 mM sodium pyrophosphate, 1 mM β-glycerophosphate, 1 mM sodium orthovanadate) supplemented with protease inhibitor cocktail (Sigma-Aldrich). Lysates were incubated end-over-end with GFP-TrapA beads (ChromoTek) overnight at 4 °C. Immunocomplexes were subsequently washed ten times with buffers containing a decreasing amount of NaCl and Tween 20 and incubated with sample buffer 2 X. The eluted proteins were resolved by SDS-PAGE and processed for western blot analysis or MS.

HEK293T cells were transfected as described above and drebrin constructs were immunoprecipitated with GFP-trap resin following the same protocol used for the SH-SY5Y cells. The eluded samples were resolved by SDS-PAGE and processed for western blot analysis to visualize the co-precipitated LRRK2 using Flag antibodies.

#### LC-MS/MS and data analysis

Three biological replicates of GFP-trap purifications of SH-SY5Y cells were processed for the proteomics experiments. Gels were stained with Colloidal Coomassie Brilliant Blue (0.25% Brilliant Blue R-250, 40% ethanol, 10% acetic acid in milli-Q water) for at least 1 hour and then rinsed in destaining solution (10% isopropanol, 10% acetic acid in milli-Q water). Gel slices were cut into small pieces and subjected to reduction with dithiothreitol (DTT 10 mM in 50 mM NH4HCO3, for 1 h at 56 °C), alkylation with iodoacetamide (55 mM in 50 mM NH4HCO3, for 45 min at RT and in the dark), and in-gel digestion with sequencing grade modified trypsin (Promega, 12.5 ng/μL in 50 mM NH4HCO3). Samples were analyzed with a LTQ-Orbitrap XL mass spectrometer (Thermo Fisher Scientific) coupled to a HPLC UltiMate 3000 (Dionex – Thermo Fisher Scientific) through a nanospray interface. Peptides were separated at a flow rate of 250 nL/min using an 11-cm-long capillary column (PicoFrit, 75 μm ID, 15 μm tip, New Objective) packed in house with C18 material (Aeris Peptide 3.6 μm XB C18; Phenomenex). A linear gradient of acetonitrile/0.1% formic acid from 3 to 40% was used for peptide separation and the instrument operated in a data dependent acquisition mode with a Top4 method (one full MS scan at 60,000 resolution in the Orbitrap, followed by the acquisition in the linear ion trap of the MS/MS spectra of the four most intense ions). Two technical replicates were acquired for each biological replicate. Raw data files were analyzed using MaxQuant and Andromeda software package (Tyanova et al., 2016) and searched against the human section of the UniProt database (version September 2020, 75093 entries) concatenated with a database of common contaminants found in proteomic experiments. Trypsin was selected as the digesting enzyme with up to two missed cleavages allowed, carbamidomethylation of cysteine residues was set as a fixed modification and methionine oxidation as a variable modification. A search against a randomized database was used to assess the false discovery rate (FDR), and data were filtered to remove contaminants and reverse sequences and keep into account only proteins identified with at least two peptides and a FDR ≤0.01, both at peptide and protein level.

Intensity values were retrieved for 350 proteins. A single step of data QC was applied to the intensity values: proteins whose intensity value was 0.00 for both the 2 technical replicates in at least 1 of the 3 pull-down experiments performed with LRRK2-GFP were removed. This is a stringent QC step, but it was implemented to remove proteins with no triplicate data available for the LRRK2 pull-down as this could have occurred because of genuine low levels of pull-down (values close to the limit of detection, missing not at random values) or because of technical issues with the single sample (loss/poor quality of sample, missing at random values). QC reduced the number of proteins in the analysis from 350 to 269 entries.

Intensity data were then processed according to Aguilan et al., 2020. Briefly, intensity data were log2 transformed and normalized to the median of all proteins within the same experiment. Missing data (0.00 intensity values) were considered missing not at random thus they were imputed via probabilistic minimum imputation (random values within less than 2.5 standard deviations from the distribution of intensity values per the single experiment, 0.3 max variability) (Aguilan et al., 2020; Lazar et al., 2016).

Finally, fold change (GFP-LRRK2 vs GFP and GFP-LRRK2 BDNF treated vs GFP-LRRK2 untreated) and associated p-value (two-tailed, paired t-test) were calculated and visualized.

### Phosphoproteomics analysis

#### Protein processing for MS

Striata from 8 weeks mice were dissected and rapidly homogenized in four volumes of ice-cold Buffer A (0.32 M sucrose, 5 mM HEPES, pH 7.4, 1 mM MgCl_2_, 0.5 mM CaCl_2_) supplemented with Halt protease and phosphatase inhibitor cocktail (Thermo Fisher Scientific) using a Teflon homogenizer (12 strokes). Homogenized brain extract was centrifuged at 1400 × *g* for 10 min. Supernatant (S1) was saved and pellet (P1) was homogenized in buffer A with a Teflon homogenizer (five strokes). After centrifugation at 700 × *g* for 10 min, the supernatant (S1’) was pooled with S1. Pooled S1 and S1′ were centrifuged at 13,800 × *g* for 10 min to the crude synaptosomal pellet (P2). The crude synaptosomal pellet (P2) was homogenized in buffer B (0.32 M sucrose, 6 mM Tris, pH 8.0) supplemented with protease and phosphatase inhibitors cocktail with a Teflon homogenizer (five strokes) and was carefully loaded onto a discontinuous sucrose gradient (0.8 M/1 M/1.2 M sucrose solution in 6 mM Tris, pH 8.0) with a Pasteur pipette, followed by centrifugation in a swinging bucket rotor for 2 hr at 82,500 g. The synaptic plasma membrane fraction (SPM) in the interphase between 1 M and 1.2 M sucrose fractions was collected using a syringe and transferred to clean ultracentrifuge tubes. 6 mM Tris buffer was added to each sample to adjust the sucrose concentration from 1.2 M to 0.32 M and the samples were centrifuged in a swinging bucket rotor at 200,000 × *g* for 30 min. The supernatant was removed and discarded. Added to the pellets the lysis solution containing 12 mM sodium deoxycholate, 12 mM sodium lauroyl sarcosinate, 10 mM TCEP, 40 mM CAA, and phosphatase inhibitor cocktail (Millipore-Sigma) in 50 mM Tris·HCl, pH 8.5, and incubated 10 min at 95 °C with vigorous shaking. Sonicated the pellets several times with a sonicator probe and boiled again for 5 min. Centrifuged at 16,000 × *g* for 10 min to remove the debris and collected supernatant. The samples were diluted fivefold with 50 mM triethylammonium bicarbonate and analyzed by BCA to determine protein concentration. The samples were then normalized to 300 μg protein in each and digested with 6 μg Lys-C (Wako) for 3 hr at 37 °C. 6 μg trypsin was added for overnight digestion at 37 °C. The supernatants were collected and acidified with trifluoroacetic acid (TFA) to a final concentration of 1% TFA. Ethyl acetate solution was added at a 1:1 ratio to the samples. The mixture was vortexed for 2 min and then centrifuged at 16,000 × *g* for 2 min to obtain aqueous and organic phases. The organic phase (top layer) was removed, and the aqueous phase was collected, dried completely in a vacuum centrifuge, and desalted using Top-Tip C18 tips (Glygen) according to manufacturer’s instructions. The samples were dried completely in a vacuum centrifuge and subjected to phosphopeptide enrichment using PolyMAC Phosphopeptide Enrichment kit (Tymora Analytical) according to manufacturer’s instructions, and the eluted phosphopeptides dried completely in a vacuum centrifuge.

#### LC-MS/MS analysis

The full phosphopeptide sample was dissolved in 10.5 μL of 0.05% trifluoroacetic acid with 3% (vol/vol) acetonitrile and 10 μL of each sample was injected into an Ultimate 3000 nano UHPLC system (Thermo Fisher Scientific). Peptides were captured on a 2 cm Acclaim PepMap trap column and separated on a 50 cm column packed with ReproSil Saphir 1.8 μm C18 beads (Dr. Maisch GmbH). The mobile phase buffer consisted of 0.1% formic acid in ultrapure water (buffer A) with an eluting buffer of 0.1% formic acid in 80% (vol/vol) acetonitrile (buffer B) run with a linear 90 min gradient of 6–30% buffer B at a flow rate of 300 nL/min. The UHPLC was coupled online with a Q-Exactive HF-X mass spectrometer (Thermo Fisher Scientific). The mass spectrometer was operated in the data-dependent mode, in which a full-scan MS (from m/z 375–1,500 with the resolution of 60,000) was followed by MS/MS of the 15 most intense ions (30,000 resolution; normalized collision energy - 28%; automatic gain control target (AGC) - 2E4, maximum injection time - 200ms; 60 sec exclusion).

#### LC-MS data processing

The raw files were searched directly against the mouse database with no redundant entries, using Byonic (Protein Metrics) and Sequest search engines loaded into Proteome Discoverer 2.3 software (Thermo Fisher Scientific). MS1 precursor mass tolerance was set at 10 ppm, and MS2 tolerance was set at 20 ppm. Search criteria included a static carbamidomethylation of cysteines (+57.0214 Da), and variable modifications of phosphorylation of S, T, and Y residues (+79.996 Da), oxidation (+15.9949 Da) on methionine residues, and acetylation (+42.011 Da) at the N terminus of proteins. Search was performed with full trypsin/P digestion and allowed a maximum of two missed cleavages on the peptides analyzed from the sequence database. The false-discovery rates of proteins and peptides were set at 0.01. All protein and peptide identifications were grouped, and any redundant entries were removed. Only unique peptides and unique master proteins were reported.

#### Label-free quantitation analysis

All data were quantified using the label-free quantitation node of Precursor Ions Quantifier through the Proteome Discoverer v2.3 (Thermo Fisher Scientific). For the quantification of phosphoproteomic data, the intensities of phosphopeptides were extracted with initial precursor mass tolerance set at 10 ppm, minimum number of isotope peaks as 2, maximum ΔRT of isotope pattern multiplets – 0.2 min, PSM confidence FDR of 0.01, with hypothesis test of ANOVA, maximum RT shift of 5 min, pairwise ratio-based ratio calculation, and 100 as the maximum allowed fold change. For calculations of fold-change between the groups of proteins, total phosphoprotein abundance values were added together, and the ratios of these sums were used to compare proteins within different samples.

#### Bioinformatics

The LRRK2 interactome was constructed following the procedure reported in Zhao et al., 2023. Briefly, protein interactions reported for LRRK2 in peer-reviewed literature were considered if the interaction was replicated in at least 2 different papers or identified with 2 different interaction detection methods. The obtained LRRK2 protein interaction network was analyzed in Zhao et al., 2023 to identify topological clusters using the FastGreed R Package (based on degree centrality). Here we report one of the identified clusters that is enriched for synaptic functions. Functional enrichment was performed using g:Profiler, g:GOSt (https://biit.cs.ut.ee/gprofiler/gost) (Raudvere et al., 2019) with Gene Ontology Biological Processes (GO-BPs) and SynGO (https://www.syngoportal.org/).

### Statistical analysis

Statistical analyses and plotting were performed with GraphPad-Prism 9. Unpaired, two-tailed t-test or Shapiro-Wilk test were used for experiments comparing two groups; one-way ANOVA was used for experiments comparing three or more groups; two-way ANOVA was used when multiple groups and two factors (i.e. genotype and treatment) were to be compared. Šídák’s multiple comparisons test was used when determining statistical significance for comparisons between groups.

## Data Availability

The data supporting the findings of this study are available within the article and its supplementary material. Protein composition of the LRRK2 cluster enriched for synaptic functions can be obtained from Zhao et al., 2023. The following previously published dataset was used: MakariousMB LeonardH NallsMA 2025Novel Parkinson’s Disease Genetic Risk Factors Within and Across European PopulationsGitHubGP2-EUR-metaGWAS

## Additional files

MDAR checklist

Supplementary file 1. Quantitative proteomic analysis of LRRK2-associated proteins under basal and BDNF-stimulated conditions.

Supplementary file 2. Functional enrichment and semantic classification of LRRK2-associated proteins involved in actin-cytoskeleton and synaptic processes.

Supplementary file 3. Comparative phosphoproteomic analysis of saline-treated LRRK2 G2019S and C57BL/6 mouse samples.
