# Supplementary material for: LRRK2 regulates synaptic function through modulation of actin cytoskeletal dynamics
Source: eLife. 2026 Aug 6;13:RP95987. doi: 10.7554/eLife.95987 (PMC13446905; doi:10.7554/eLife.95987)
Supplement: Supplementary file 3 [file elife-95987-fig1-data2.zip › Figure1-Source Data 2/Fig1.J.pdf]

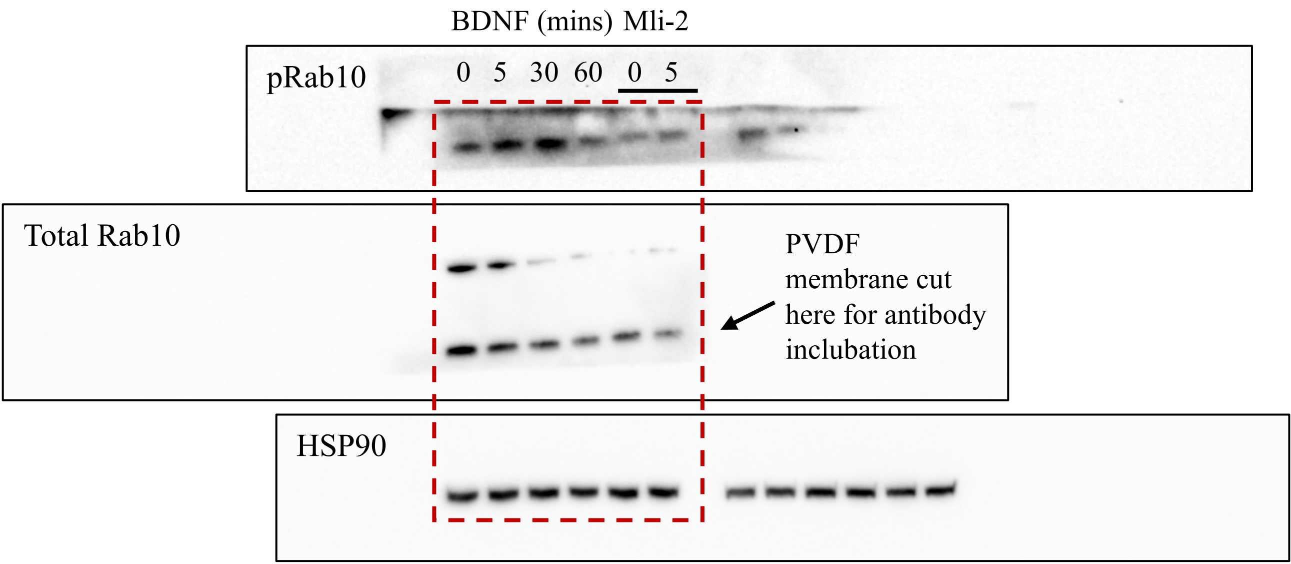
BDNF (mins) Mli-2
pRab10
0 5 30 60 0 5
Total Rab10
PVDF membrane cut here for antibody inclubation
HSP90
