# Supplementary material for: LRRK2 regulates synaptic function through modulation of actin cytoskeletal dynamics
Source: eLife. 2026 Aug 6;13:RP95987. doi: 10.7554/eLife.95987 (PMC13446905; doi:10.7554/eLife.95987)
Supplement: Supplementary file 5 [file elife-95987-fig1-figsupp1-data2.zip › Figure 1-figure supplement 1-Source Data 2/Fig1.S1.A.pdf]

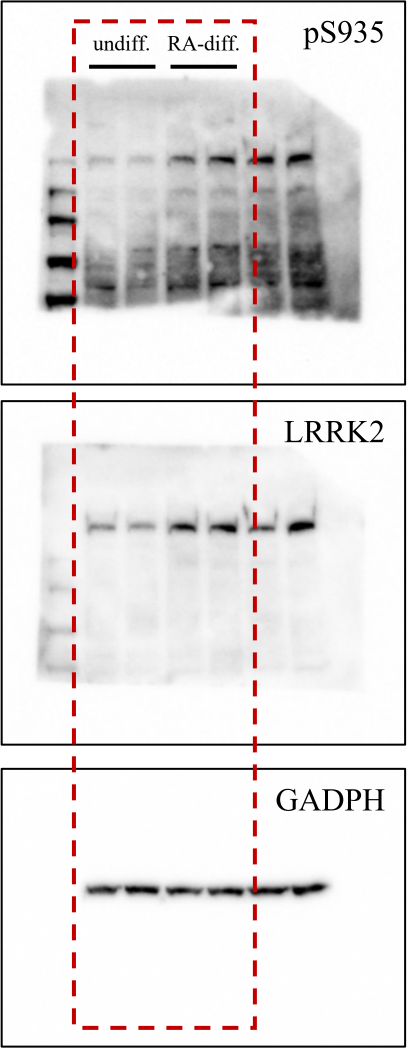

pS935
undiff.
RA-diff.
LRRK2
GADPH
