# Supplementary material for: LRRK2 regulates synaptic function through modulation of actin cytoskeletal dynamics
Source: eLife. 2026 Aug 6;13:RP95987. doi: 10.7554/eLife.95987 (PMC13446905; doi:10.7554/eLife.95987)
Supplement: Supplementary file 5 [file elife-95987-fig1-figsupp1-data2.zip › Figure 1-figure supplement 1-Source Data 2/Fig1.S1.B.pdf]

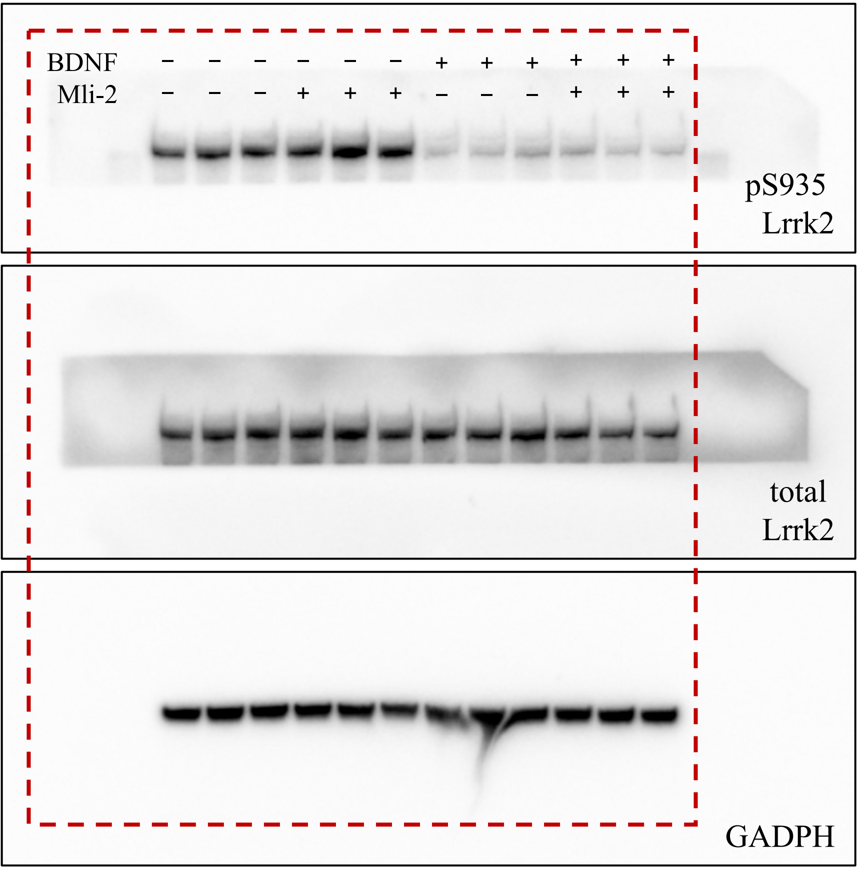

BDNF – – – – – – + + + + + +
Mli-2 – – – + + + – – – + + +
pS935 Lrrk2
total Lrrk2
GADPH
