# Supplementary material for: LRRK2 regulates synaptic function through modulation of actin cytoskeletal dynamics
Source: eLife. 2026 Aug 6;13:RP95987. doi: 10.7554/eLife.95987 (PMC13446905; doi:10.7554/eLife.95987)
Supplement: Supplementary file 5 [file elife-95987-fig1-figsupp1-data2.zip › Figure 1-figure supplement 1-Source Data 2/Fig1.S1.C.pdf]

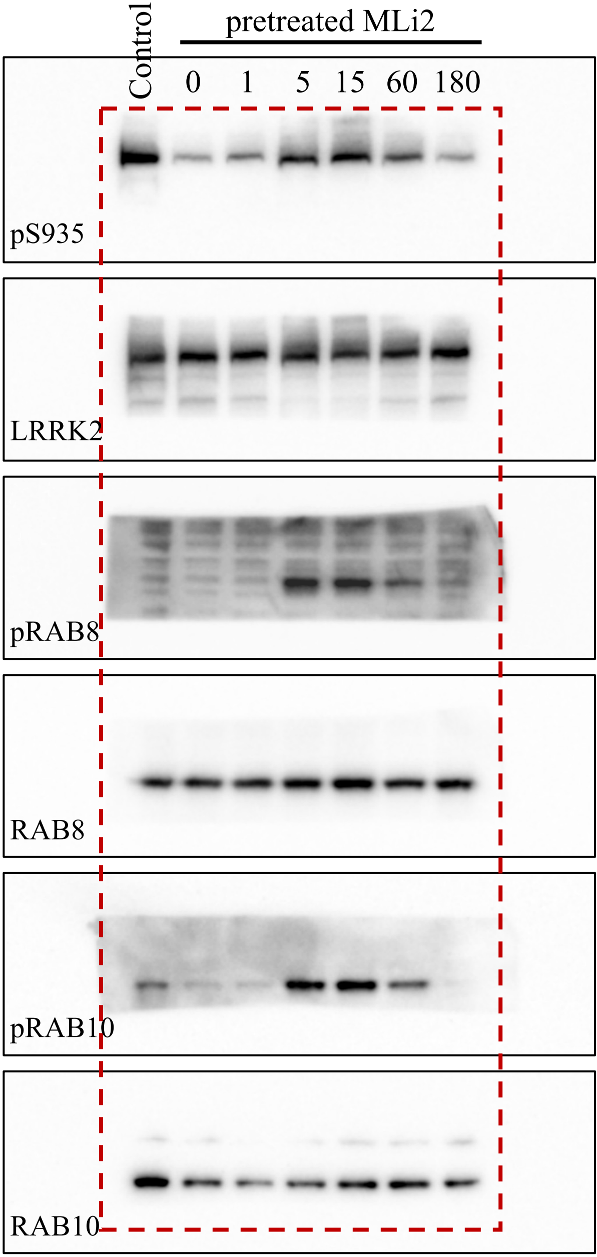
Control
pretreated MLi2
0
1
5
15
60
180
pS935
LRRK2
pRAB8
RAB8
pRAB10
RAB10
