# Supplementary material for: LRRK2 regulates synaptic function through modulation of actin cytoskeletal dynamics
Source: eLife. 2026 Aug 6;13:RP95987. doi: 10.7554/eLife.95987 (PMC13446905; doi:10.7554/eLife.95987)
Supplement: Supplementary file 7 [file elife-95987-fig2-data2.zip › Figure2-Source Data 2/Fig2.G.pdf]

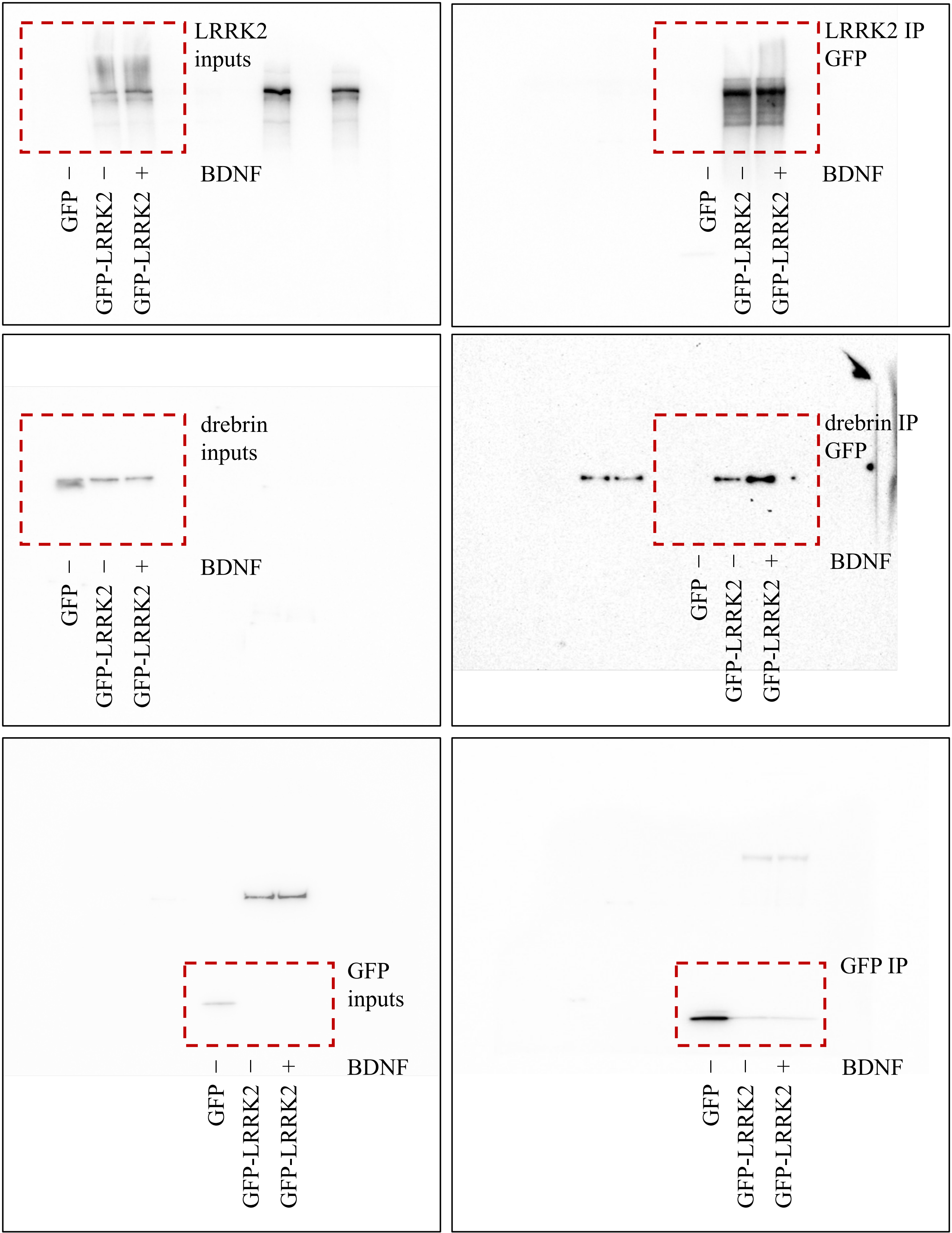
LRRK2
inputs
BDNF
GFP
GFP-LRRK2
GFP-LRRK2
LRRK2 IP
GFP
drebrin
inputs
drebrin IP
GFP
GFP
inputs
GFP IP
