# Supplementary material for: LRRK2 regulates synaptic function through modulation of actin cytoskeletal dynamics
Source: eLife. 2026 Aug 6;13:RP95987. doi: 10.7554/eLife.95987 (PMC13446905; doi:10.7554/eLife.95987)
Supplement: Supplementary file 9 [file elife-95987-fig2-figsupp1-data2.zip › Figure 2-figure supplement 1-Source Data 2/Fig2.S1.B.pdf]

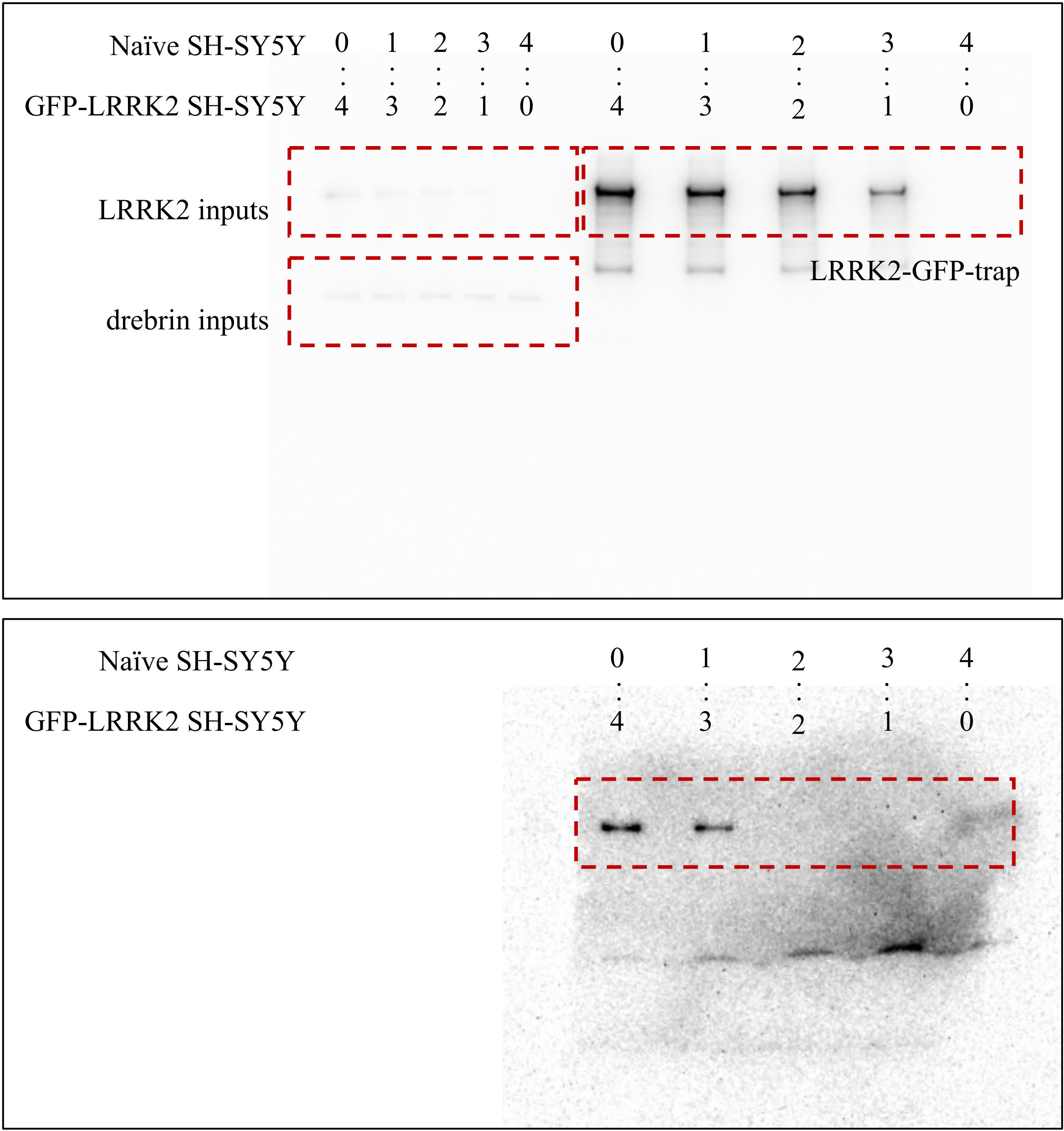
Naïve SH-SY5Y 0 : 4 1 : 3 2 : 2 3 : 1 4 : 0 0 : 4 1 : 3 2 : 2 3 : 1 4 : 0
GFP-LRRK2 SH-SY5Y
LRRK2 inputs
drebrin inputs
LRRK2-GFP-trap
Naïve SH-SY5Y 0 : 4 1 : 3 2 : 2 3 : 1 4 : 0
GFP-LRRK2 SH-SY5Y
