# Supplementary material for: LRRK2 regulates synaptic function through modulation of actin cytoskeletal dynamics
Source: eLife. 2026 Aug 6;13:RP95987. doi: 10.7554/eLife.95987 (PMC13446905; doi:10.7554/eLife.95987)
Supplement: Supplementary file 9 [file elife-95987-fig2-figsupp1-data2.zip › Figure 2-figure supplement 1-Source Data 2/Fig2.S1.C.pdf]

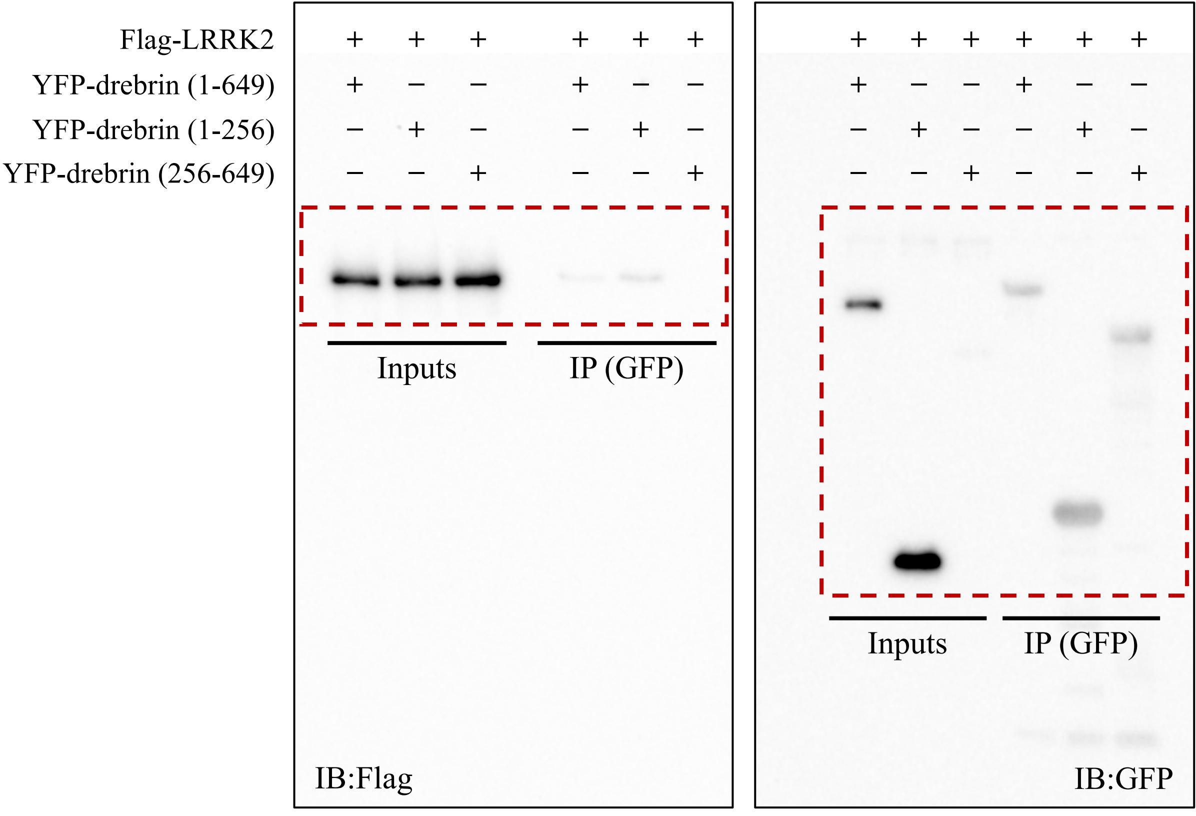
Flag-LRRK2
YFP-drebrin (1-649)
YFP-drebrin (1-256)
YFP-drebrin (256-649)
Inputs
IP (GFP)
IB:Flag
Inputs
IP (GFP)
IB:GFP
