# Supplementary material for: LRRK2 regulates synaptic function through modulation of actin cytoskeletal dynamics
Source: eLife. 2026 Aug 6;13:RP95987. doi: 10.7554/eLife.95987 (PMC13446905; doi:10.7554/eLife.95987)
Supplement: Supplementary file 11 [file elife-95987-fig4-data2.zip › Figure4-Source Data 2/Fig4.C.pdf]

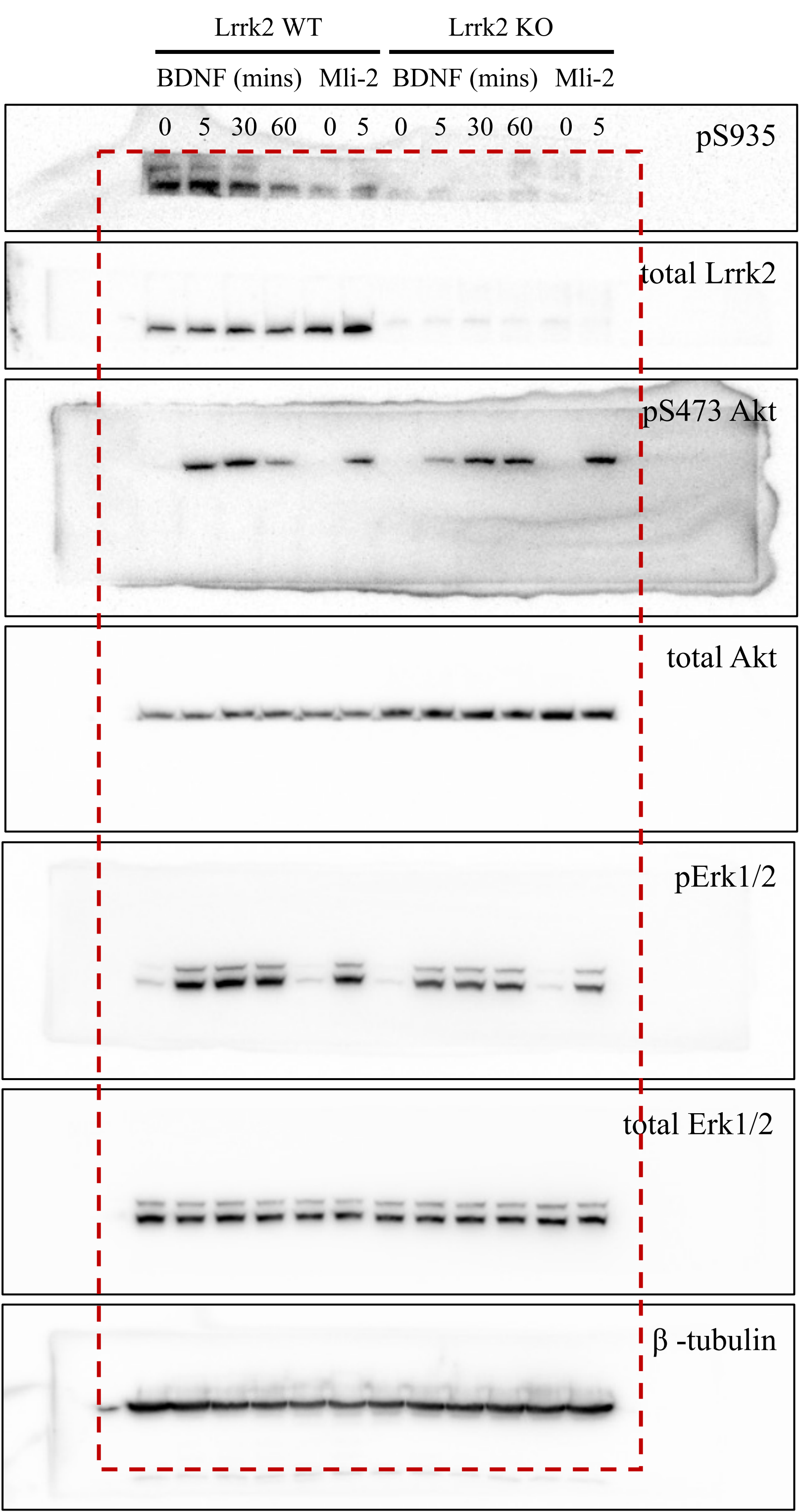
Lrrk2 WT
Lrrk2 KO
BDNF (mins)
Mli-2
BDNF (mins)
Mli-2
0 5 30 60 0 5 0 5 30 60 0 5
pS935
total Lrrk2
pS473 Akt
total Akt
pErk1/2
total Erk1/2
β -tubulin
