# Supplementary material for: LRRK2 regulates synaptic function through modulation of actin cytoskeletal dynamics
Source: eLife. 2026 Aug 6;13:RP95987. doi: 10.7554/eLife.95987 (PMC13446905; doi:10.7554/eLife.95987)
Supplement: Supplementary file 11 [file elife-95987-fig4-data2.zip › Figure4-Source Data 2/Fig4.A.pdf]

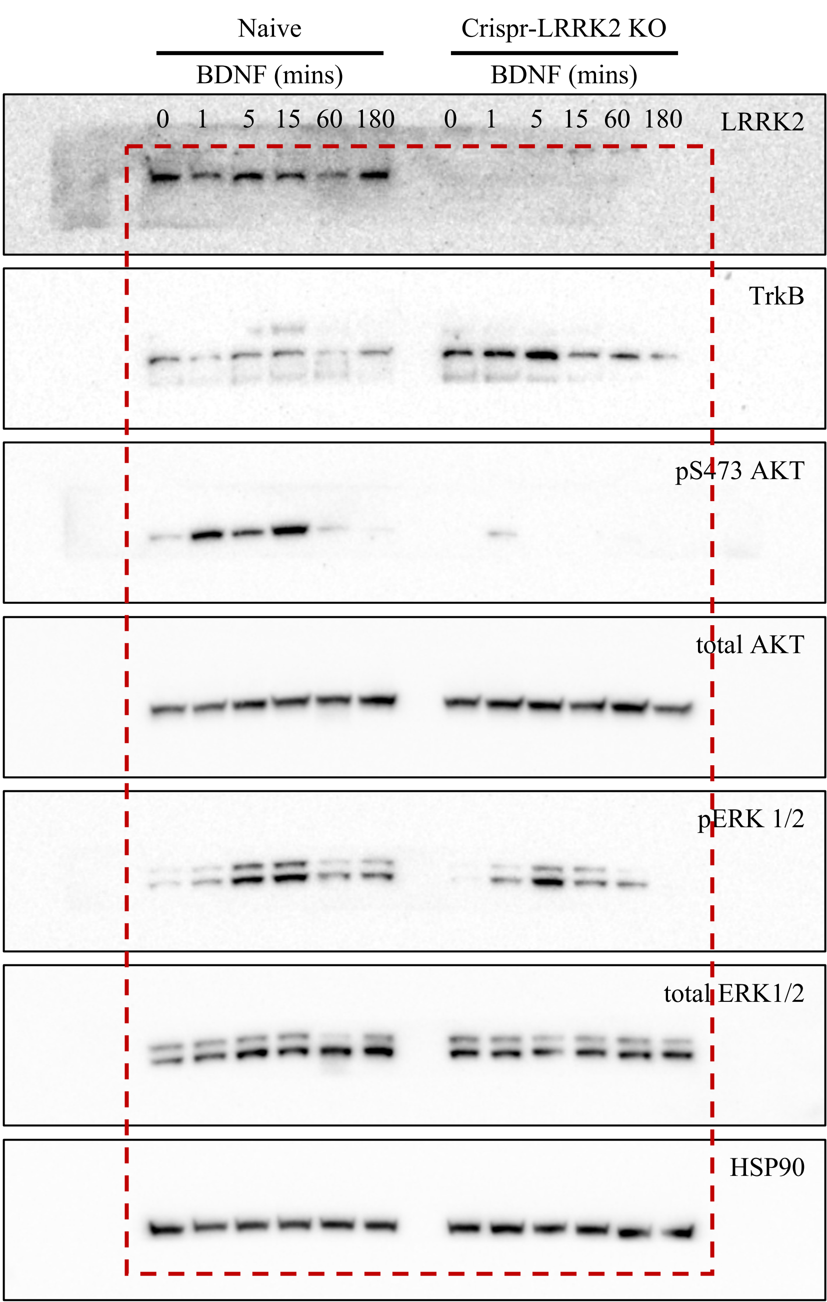
Naive
Crispr-LRRK2 KO
BDNF (mins)
BDNF (mins)
0 1 5 15 60 180
0 1 5 15 60 180
LRRK2
TrkB
pS473 AKT
total AKT
pERK 1/2
total ERK1/2
HSP90
