# Supplementary material for: LRRK2 regulates synaptic function through modulation of actin cytoskeletal dynamics
Source: eLife. 2026 Aug 6;13:RP95987. doi: 10.7554/eLife.95987 (PMC13446905; doi:10.7554/eLife.95987)
Supplement: Supplementary file 13 [file elife-95987-fig4-figsupp1-data2.zip › Figure 4-figure supplement 1-Source Data 2/Fig4.S1.pdf]

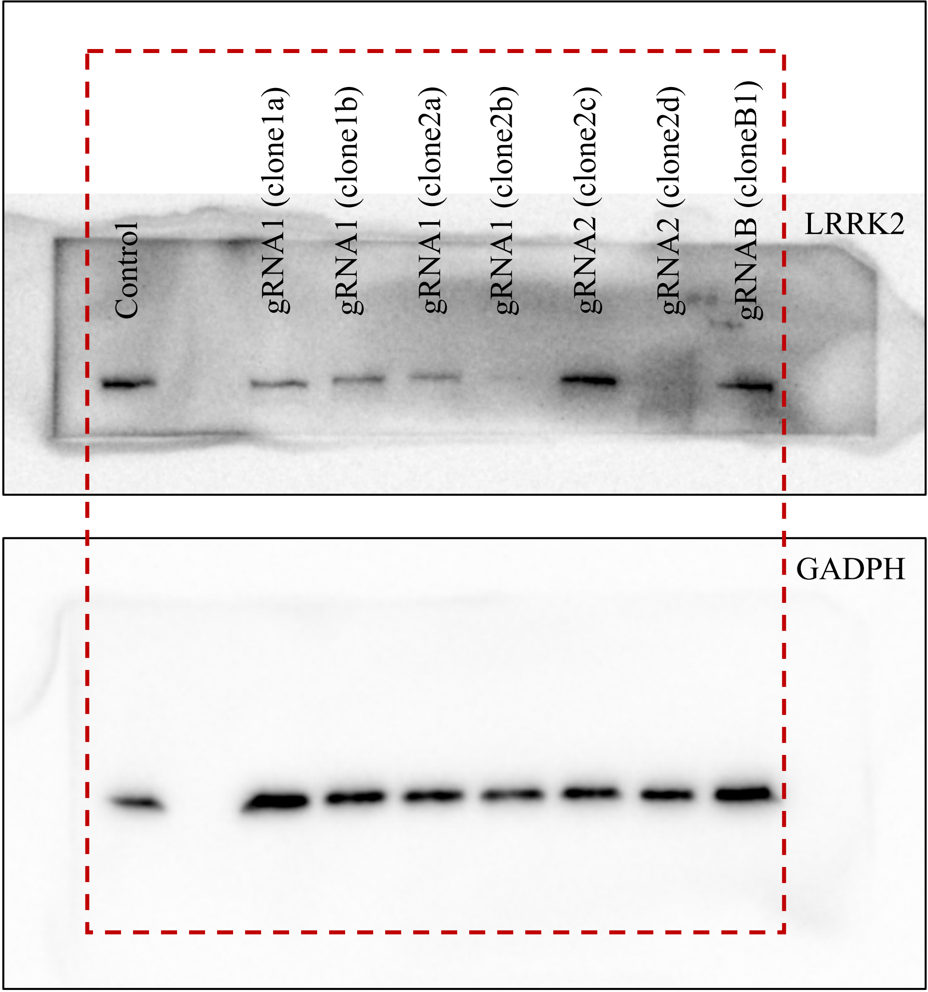
Control
gRNA1 (clone1a)
gRNA1 (clone1b)
gRNA1 (clone2a)
gRNA1 (clone2b)
gRNA2 (clone2c)
gRNA2 (clone2d)
gRNAB (cloneB1)
LRRK2
GADPH
