# Supplementary material for: LRRK2 regulates synaptic function through modulation of actin cytoskeletal dynamics
Source: eLife. 2026 Aug 6;13:RP95987. doi: 10.7554/eLife.95987 (PMC13446905; doi:10.7554/eLife.95987)
Supplement: Supplementary file 15 [file elife-95987-fig5-data2.zip › Figure5-Source Data 2/FigS1.B_4months.pdf]

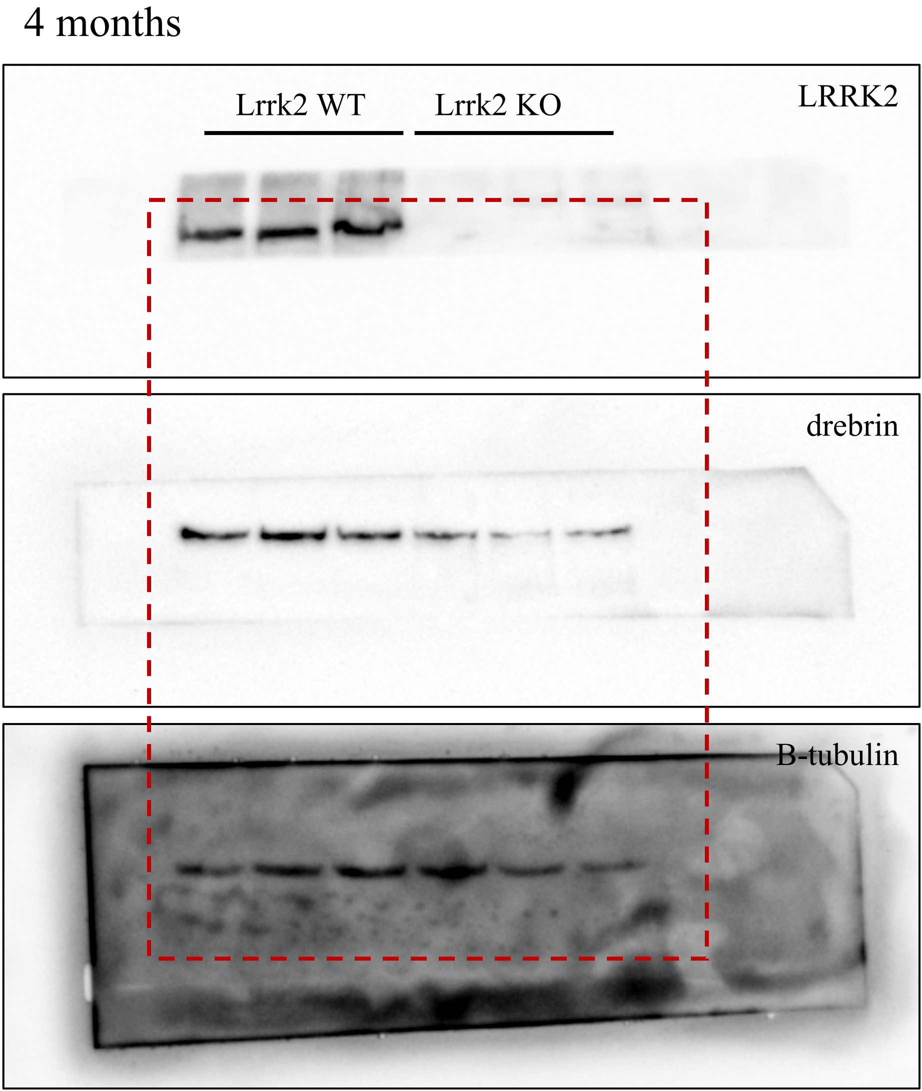
4 months
Lrrk2 WT
Lrrk2 KO
LRRK2
drebrin
B-tubulin
