# Supplementary material for: LRRK2 regulates synaptic function through modulation of actin cytoskeletal dynamics
Source: eLife. 2026 Aug 6;13:RP95987. doi: 10.7554/eLife.95987 (PMC13446905; doi:10.7554/eLife.95987)
Supplement: Peer review history

# Editor report: eLife Assessment

**DOI:** 10.7554/eLife.95987.4.sa0

**Authors:** Andrew B West

**Affiliations:** Duke University United States

**Keywords:** Important, Solid

This **important** study identifies a physiologically relevant interaction between LRRK2 and drebrin, an actin-binding protein crucial for neuronal structure. A **solid** body of evidence, including multiple cell models, highlights the complexities of how modifiers like BDNF intersect with LRRK2-kinase dependent function, and that many modifiers between AKT and LRRK2 in different cellular pathways are yet to be identified and understood.

# Referee report: Reviewer #1 (Public review)

**DOI:** 10.7554/eLife.95987.4.sa1

Summary:

LRRK2 protein is familially linked to Parkinson's disease by the presence of several gene variants that confer a gain-of-function effect on LRRK2 kinase activity.

The authors examine the effects of BDNF stimulation in immortalized neuron-like cells, cultured mouse primary neurons, hIPSC-derived neurons, and brain tissue from genetically modified mice. They examine a LRRK2 regulatory phosphorylation residue, LRRK2 binding relationships, other kinase phosphorylation status, and measures of synaptic structure and function.

Strengths:

The study addresses an important research question: how does a PD-linked protein interact with other proteins, and contribute to responses to a well-characterized neuronal signalling pathway involved in the regulation of synaptic function and cell health.

They employ a range of models and techniques to convincingly demonstrate that BDNF stimulation alters LRRK2 phosphorylation at pS935 and binding to many proteins. Several independent data sets lead to some exciting conclusions.

In this re-revised manuscript, some aspects are very convincing and well validated e.g., drebrin binding to LRRK2, increased by BDNF, and reduced LRRK2 protein levels in young (but not mature) drebrin KO mice. A phosphoproteomic analysis of PD mutant Knock-in mouse brain is included. Overall, the links between LRRK2, LRRK2 activity, and the changes to synaptic molecules, structures, and activity are intriguing.

Weaknesses:

Enthusiasm for the title claim that "LRRK2 regulates synaptic function through BDNF signalling" is tempered by disconnected results across different model systems and inconsistent alterations upon kinase phosphorylation in SHSY5Y cell line and primary neurons. Exciting conclusions are sometimes not consistently supported by the data and/or only conducted in one of the models.

BDNF increasing pS935 LRRK2 is quite well supported in cell lines, as is BDNF regulation of derbrin-LRRK2 binding. However, there is a lack of connection between this result and subsequent alterations to LRRK2 substrates e.g., phosphorylation of Rab GTPases, especially in neurons. Interesting omic data sets are provided, but with very little or no validation. For example, only drebrin protein was assessed in BDNF treatment omic, and the phosphoproteomic analysis of PD mutant Knock-in mouse is stand alone with no validation and G2019S is not explored elsewhere in the study.

The major disconnect this reviewer struggles with is the conclusion that the quite clear data in SHSY5Y cells is the same as that from neurons regarding BDNF / LRRK2 and ERK / Akt. It seems they are not.

ERK and Akt phosphorylation by BDNF is absent in CRISPR KO SHSY5Y cells.

This conclusion is at odds with interpretation of neuronal data. To explain; in div14 neurons, BDNF's transient increase in pLRRK2 is seen and strongly prevented by MLi2. BDNF also increased pAkt & pERK1&2 in WT... but also in LRRK2 KO. Furthermore, this happened in the presence of MLi2 in WT despite no pLRRK2 increase. While the 5min BDNF induced increase to pAkt appears reduced in LKO, the same time BDNF in LKO with MLi2 is as high as WT (in these unquantified examples) and ERK is almost identical. This is described as "significantly reduced" but I see no replicates or quantification, and face value assessment of the blot argues against this.

Thus, there is little or no evidence supporting that LRRK2 activity is involved in BDNF-stimulated increases in pAkt or pERK, upstream, in neurons as neither Mli2 nor KO prevented this.

Synapse markers increased in WT neuron with BDNF treatment which did not happen in LKO neurons. So this process requires pLRRK2, but is unrelated to pAkt or pERK (which do still go up with BDNF in KO)? Similarly, an increase in synaptic activity in WT hiPSC neurons in response to BDNF seems lost in LRRK2 KO hiPSC neurons, although their activity is already increased and depending on the age of the cells the effects were different. Both of these experiments lack supporting evidence by other measures e.g., LRRK2 inhibition effects on BDNF-induced increases in WT and parallel biochemistry of p'd LRRK2, Akt, ERK in WT & KO.

LRRK2 activating Akt1 has been published before (e.g., Ohta 2011 - not cited), but Ohta also conclude that LRRK2 gain of function mutations (more LRRK2 kinase activity) were associated with a reduced ability of LRRK2 to bind AND phosphorylate Akt at the same residue, in contradiction to the mechanism proposed here? This should be discussed. Here the authors also conclude Akt is Upstream of LRRK2. However, it appears from the data here in neurons that pLRRK2 increases in response to BDNF are separate from BDNF signalling to Akt.

Of note, in comparison to bTubulin control, LKO total Akt levels appear consistently higher in this single example blot; a large increase in Akt would skew the ratio down, while absolute levels of pAkt (probably the most important matter for an active enzyme - what is the ratio against total protein stain) are similar or increased. These are major problems for the conclusions as presented.

BDNF increased mEPSC frequency in hIPSC neurons; which didn't happen in LKO, which already had high frequency. Earlier in the manuscript BDNF is shown to alter synapse number in WT but not LKO mouse neurons, but no increase in synapse number was seen following BDNF treatment in any WT or LKO hiPSC neurons +/- BDFN.

If we are to assume that the WT neurons have LRRK2 (not demonstrated), and that LRRK2 KO neurons have similar drebrin (not demonstrated) it is unclear how to interpret this result in the model of BDNF-LRRK2 being upstream of pERK/Akt. There is no evidence that the BDNF increase in WT is blocked by LRRK2 inhibition, nor has it been associated with changes (or not) to pAkt or ERK1, which would be expected in both WT and KO based on Figure 4C.

There are many reports of acute and longer term BDNF application increasing event frequency in brain slices & primary neurons. Overexpression of BDNF in NPCs has also been shown to increase synapse function in hiPSC neurons derived from them. Here, BDNF has an effect on frequency in only one 6 comparisons (3 timepoints, two lines). Is it not concerning that expected BDNF effects occur at only one time point in WT, and that generally a lack of effect is more common both in WT and LKO... is this due to slow appearance of TrkB receptors and degeneration at 90 days?

There are no other data provided to show that BDNF was having a consistent expected effect in human neurons (pAkt, pLRRK2 etc etc), and there is little to link between this data and that in previous figures of the study.

The discussion of some of the weaknesses is mostly fair, asides the disparities noted above which are not.

# Referee report: Reviewer #2 (Public review)

**DOI:** 10.7554/eLife.95987.4.sa2

The data show that BDNF regulates the PD-associated kinase LRRK2, they place LRRK2 within well-described BDNF pathways biochemically, and they show that LRRK2 can play a role mediating BDNF-driven synaptic outcomes at excitatory synapses. The chief strength is that the data provide a potential focal point for multiple observations that have been made across many labs. The findings will be of broad interest because LRRK2 has emerged as a protein that is likely to be part of Parkinson's pathology and its normal and pathological actions remain poorly understood.

A major strength of the study is the multiple approaches that were used (biochemistry, bioinformatics, light and electron microscopy and electrophysiology) across different experimental models (cells, primary neurons, human neurons, mice) to identify and examine the impact of BDNF on LRRK2 signaling and functions. Noteworthy is also the employment of LRRK2KO preparations to validate outcomes and to place LRRK2 actions up or downstream.

The demonstration that LRRK2 and drebrin interact directly is important and suggests that other interacting proteins identified biochemically and bioinformatically in the paper will be important to pursue.

# Author comment: Author response

**DOI:** 10.7554/eLife.95987.4.sa3

**Authors:** Giulia Tombesi, Shiva Kompella, Giulia Favetta, Chuyu Chen, Marta Ornaghi, Yibo Zhao, Ester Morosin, Martina Sevegnani, Adriano Lama, Antonella Marte, Ilaria Battisti, Lucia Iannotta, Nicoletta Plotegher, Laura Civiero, Franco Onofri, Britta Eickholt, Giovanni Piccoli, Giorgio Arrigoni, Dayne Beccano-Kelly, Claudia Manzoni, Loukia Parisiadou, Elisa Greggio

**Affiliations:** Department of Biology, University of Padova, Italy Padova Italy; School of Pharmacy and UK Dementia Research Institute, Cardiff University UK Cardiff United Kingdom; Department of Biology, University of Padova, Italy Padova Italy; Feinberg School of Medicine, Northwestern University Chicago United States; Institute for Biochemistry, Charite Universitatsmedizin Berlin, Berlin, Germany Berlin Germany; School of Pharmacy, University College London, UK London United Kingdom; Department of Biology, University of Padova, Italy Padova Italy; CIBIO, University of Trento, Italy Trento Italy; CIBIO, University of Trento Trento Italy; University of Genova Genova Italy; University of Padua Padova Italy; Department of Biology, University of Padova, Italy Padova Italy; Department of Biology, University of Padova, Italy Padova Italy; Department of Biology, University of Padova, Italy Padova Italy; University of Genova Genova Italy; Charité Universitätsmedizin Berlin Berlin Germany; CIBIO, University of Trento, Italy Trento Italy; Department of Biomedical Sciences, University of Padova, Italy Padova Italy; School of Pharmacy and UK Dementia Research Institute, Cardiff University UK Cardiff United Kingdom; School of Pharmacy, University College London, UK London United Kingdom; Feinberg School of Medicine, Northwestern University Chicago United States; University of Padua Padova Italy

The following is the authors’ response to the previous reviews

**eLife Assessment**

This important work begins to understand how BDNF regulates the phosphorylation and activity of LRRK2. The overall strength of evidence has been assessed as compelling, though some claims are only partially supported. The work will be of interest for those that might pursue specific LRRK2 interactions and mutational effects on these pathways as the work continues to develop.

We thank the editors and reviewers for the constructive feedback. We have revised the manuscript to improve clarity, strengthen statistical analysis and increase the western blot sample size in drebrin KO mice.

**Public Reviews:**

**Reviewer #1 (Public review):**

Summary:

LRRK2 protein is familially linked to Parkinson's disease by the presence of several gene variants that all confer a gain-of-function effect on LRRK2 kinase activity.

The authors examine the effects of BDNF stimulation in immortalized neuron-like cells, cultured mouse primary neurons, hIPSC-derived neurons, and brain tissue from genetically modified mice. They examine a LRRK2 regulatory phosphorylation residue, LRRK2 binding relationships, and measures of synaptic structure and function.

Strengths:

The study addresses an important research question: how does a PD-linked protein interact with other proteins, and contribute to responses to a well-characterized neuronal signalling pathway involved in the regulation of synaptic function and cell health.

They employ a range of good models and techniques to fairly convincingly demonstrate that BDNF stimulation alters LRRK2 phosphorylation and binding to many proteins. In this revised manuscript, aspects are well validated e.g., drebrin binding, but there is a disconnect between these findings and alterations to LRRK2 substrates. A convincing phosphoproteomic analysis of PD mutant Knock-in mouse brain is included. Overall the links between LRRK2, LRRK2 activity, and the changes to synaptic molecules, structures, and activity are intriguing.

We thank this Reviewer for appreciating our work including the new experiments performed during the revisions.

Weaknesses:

The data sets remain disjointed, conclusions are sweeping, and not always in line with what the data is showing. Validation of 'omics' data is light. Some inconsistencies with the major conclusions are ignored. Several of the assays employed (western blotting especially) are underpowered, findings key to their interpretation are addressed in only one or other of the several models employed, and supporting observations are lacking.

We understand the Reviewer’s points and agree that it is important to increase the sample size (animals) for western blot. In particular, we acknowledge that the initial experiments with the Dbn1 KO mice included only 3 mice, which was insufficient to draw any definitive conclusion on the effect, especially regarding pRab8 levels. In response to this, we have collected additional animals and repeated the experiment with N=7 wild-type and N=7 KO mice (2 months old). Despite a high degree of interindividual variability, we have confirmed that drebin KO mouse brains have reduced levels of pLRRK2 (Author response image 1). In the new figure 2H we included all the replicates (N=7+3 per genotype) for pLRRK2. However, we removed western blot for pRab8 because a new batch of pRab8 antibody did not yield specific results, making it impossible to reassess.

**Author response image 1.** Western blot analysis of N=7 WT and N=7 drebrin KO whole brains.

Main Conclusions of Abstract:

(1) Increase in pLRRK2 Ser935 and pRAB after BDNF in SH-SY5Y & mouse neurons

Well supported, but only for pLRRK2 in neurons, why not pERK pAkt & pRab?

The response of pERK and pAKT in neurons is shown in figure 4C. We have repeatedly tried pRab (both pRab8 and pRab10) in primary neurons but with no success. In support of the difficulty in detecting pRab in primary neurons, we are not aware of studies in the literature of western blot analysis of pRabs in primary neuronal cultures. This is likely due to the high levels of PPM1H in neurons as discussed in Berndsen et at, eLife, 2019 (PMID: 31663853).

(2) Omics Proteome remodelling of LRRK2 interactome with BDNF & different in G2019S mouse neurons.

Supports that the phosphoproteome of G2019S is different. Drebrin interaction with LRRK2 very well supported. Link between drebrin and LRRK2 activity somewhat supported (pS935 site), but the consequence (non-specific pRab8) not supported, as there is no evidence of a change in LRRK2 substrate(s).

As discussed above, we removed the pRab8 western blot in figure 2H as we could not confirm with the new set of mice and a new batch of pRab8 antibody.

(3) Golgi 1 month LKO mouse altered dendritic spines, transient at 1m not older.

Supported but very small transient change in spines, disconnected to other results (e.g., drebrin).

We agree with the Reviewer that the observed effect is modest, still we believe it is important to report. As discussed in the discussion, one plausible explanation for the limited magnitude of the effect is functional compensation by LRRK1.

(4) iPSC-derived neurons BDNF increases mEPSC frequency (transient at 70 not 50 or 90 days) in WT not KO "which appear to bypass this regulation through developmental compensation"

Weak, not clear what is being bypassed.

We reviewed the statistical analysis as described below.

Main Conclusions Based on Old and New Figure / Data:

(1) Increase in pLRRK2 Ser935 and pRAB after BDNF in SH-SY5Y & mouse neurons

Well supported, but only for pLRRK2 in neurons, why not ERK Akt & Rab?

The response of pERK and pAKT in neurons is shown in figure 4C. We have repeatedly tried pRab (both pRab8 and pRab10) in primary neurons but with no success. In support of the difficulty in detecting pRab in primary neurons, we are not aware of studies in the literature of western blot analysis of pRabs in primary neuronal cultures. This is likely due to the high levels of PPM1H in neurons as discussed in Berndsen et at, eLife, 2019 (PMID: 31663853).

(2) BDNF promotes LRRK2 interaction with "post-synaptic actin cytoskeleton components"

Tone down, only one postsynaptic validated - drebrin strong BUT CONTRADICTORY; link between drebrin and LRRK2 activity (pS935 site) supported, consequence (non-specific pRab8) broken, no evidence of change in LRRK2 substrate.

As suggested we tone down the paragraph title and changed it as follow: “BDNF stimulates LRRK2 interaction with drebrin, an actin cytoskeletal-associated protein enriched at the postsynapse”. As mentioned above, pRab8 has not been incorporated.

(3) LRRK2 G2019S striatal phosphoproteome is different from WT.

It is different. Where is link to BDNF or Drebrin?

We found that debrin S339 phosphorylation is 3.7 fold higher in G2019S KI mice as compared to WT, suggesting a potential functional connection between LRRK2 and drebrin. However, differences in phosphorylation do not necessarily translate into physiological effects so further validation is required. To test if BDNF can induce S339 drebrin phosphorylation in a LRRK2-dependent manner we plan an in vivo experiment where BDNF is acutely administered to WT vs G2019S-KI mice +/- MLi2 to control for LRRK2 dependency. This is an important experiment to establish the mechanistic link, though it will require sufficient time due to the necessary ethical authorization needed to administer BDNF in the mouse brain.

(4) BDNF signaling is impaired in Lrrk2 knockout neurons

TrkB changes seem higher in SHSY5Y. pAKT impaired, pERK not convincing. Primary neurons Akt slower but it and Erk mostly intact. MLi-2 did not block pAkt or pErk in WT or KO (higher in latter). Whatever is happening in KO, Mli-2 not really blocking effect in WT. If we are to assume that studying the KO was a means to understand LRRK2 function, the authors data should explain why we care if an effect is absent in LKO, if LRRK2 isn't doing the same job in WT?

To further support the conclusion that this effect is reproducible and dependent on LRRK2 kinase activity acting upstream of AKT and ERK signaling, we probed the membranes shown in Figure 1H for phosphorylated and total AKT and ERK1/2. Consistent with our hypothesis, the inhibition of LRRK2 with MLi-2 significantly reduced BDNF-induced AKT and ERK1/2 phosphorylation (Author response image 2).

**Author response image 2.** Western blot (same experiments as in figure 1) was performed using antibodies against phosphoThr202/185 ERK1/2, total ERK1/2 and phospho-Ser473 AKT, total AKT protein levels. Retinoic acid-differentiated SH-SY5Y cells stimulated with 100 ng/mL BDNF for 0, 5, 30, 60 mins. MLi-2 was used at 500 nM for 90 mins to inhibit LRRK2 kinase activity.

BDNF increases synaptic puncta in WT not LKO (which start higher?). Is this BDNF increase blocked by LRRK2 inhibition?

This is an important experiment that we plan to investigate in a future study.

(5) Postsynaptic structural changes in Lrrk2 knockout neurons

Golgi impregnation shows some very small spine changes at 1m. Not sustained over age. mRNA changes are very small (10% not even a fold... very weak and should be written as so). Derbrin levels reduced clearly at 1m, but probably also at 4 & 18. Underpowered, disconnected time course from the spine changes.

While differences are small they have been observed in independent sets of mice through qPCR, histology, WB and TEM, supporting the consistency of the effect, although small. For clarity we rescaled the qPCR graphs to 0.

(6) An effect on "spontaneous electrical activity" at Div70

Weak. What is so special at 70 days that means we should be confident in the differences, or be satisfied that the other time points are legitimately ignored? These are 10-11 cells from 3 cultures assayed at 3 time points but only one is presented (rest in supplement). This should be a 2 (time) or 3 way (+culture RM) ANOVA. As it stands, in WT there is a little - no activity at 50 days, little to no at 70 days, and variable to lots or none at 90. BDNF did nothing at 50 or 90 but may have at 70. In KO low activity stable at 50 & 70, tanks at 90. BDNF would seem to have a similar effect on KO at 90 as WT at 70, but as there are only 7 cells it remains inconclusive. Thus the conclusion that BDNF signalling is broken in LKO is not well supported by the ephys data, nor is the BDNF effect in WT cells (even at the 70 day time point) shown to be susceptible to LRRK2 inhibition.

We thank the Reviewer for suggesting a more comprehensive analysis of the data. Following this suggestion, we performed separate two-way ANOVAs (DIV × treatment) for WT and LRRK2 KO neurons. This analysis revealed significant main effects of DIV and BDNF treatment in WT neurons, indicating that synaptic activity increases with neuronal maturation and is globally enhanced by BDNF. In contrast, neither DIV nor BDNF treatment reached statistical significance in LRRK2KO neurons, and no DIV × treatment interaction was observed. These results indicate that BDNFdependent enhancement of synaptic activity is preserved in WT neurons but is lost in the absence of LRRK2. We have now incorporated this analysis into the main figure and removed the individual DIV50 and DIV90 plots from the supplementary material. We also revised the title of the last paragraph to reflect the outcome of this analysis and toned down our interpretation (page 12).

Furthermore, we have added a paragraph to the Discussion section highlighting the limitations of this study. These include the variability observed in protein content and phosphorylation analyses by western blot, as well as the necessity to confirm the electrophysiological findings in larger datasets, including in dopaminergic neurons.

**Reviewer #2 (Public review):**

The data show that BDNF regulates the PD-associated kinase LRRK2, they place LRRK2 within welldescribed BDNF pathways biochemically, and they show that LRRK2 can play a role mediating BDNFdriven synaptic outcomes at excitatory synapses. The chief strength is that the data provide a potential focal point for multiple observations that have been made across many labs. The findings will be of broad interest because LRRK2 has emerged as a protein that is likely to be part of Parkinson's pathology and its normal and pathological actions remain poorly understood.

We thank this Reviewer for appreciating our work and acknowledging that our findings will be of broad interest.

A major strength of the study is the multiple approaches that were used (biochemistry, bioinformatics, light and electron microscopy and electrophysiology) across different experimental models (cells, primary neurons, human neurons, mice) to identify and examine the impact of BDNF on LRRK2 signaling and functions. Noteworthy is also the employment of LRRK2KO preparations to validate outcomes and to place LRRK2 actions up or downstream.

Thank you to the Reviewer

The demonstration that LRRK2 and drebrin interact directly is important and suggests that other interacting proteins identified biochemically and bioinformatically in the paper will be important to pursue.

We agree with this statement

Some data from different models do not fit well with one another (like mouse and human neurons). This is likely due to inherent differences in the preparations. Since different experiments were carried out on the different preps, however, it is not possible to cross compare. The lack of this information is viewed more as an open question than a cause for concern.

We thank the Reviewer for raising this point. In response, we have added a new section to the Discussion explicitly addressing the limitations of the study.

**Recommendations for the authors:**

**Reviewer #1 (Recommendations for the authors):**

MLi2 pretreatment experiment is nice. They state in legends BDNF treatment prior to MLi2, they mean prior MLI2 treatment. Or MLi2 pretreatment, prior to BDNF. However, this experiment is hard to interpret as it has no control (non BDNF treated) time course following MLi2, could this be (at least in part) a rebound effect produced by relief of inhibition? This should be discussed if not addressed directly by experiments.

The non BDNF treated group represents the 0 time point. We have specified it in the figure legend. We have excluded the rephosphorylation kinetic after MLi-2 relief because pRabs increase significantly at 5 minutes, far exceeding the control levels. This observation gives us feel confidence that the effect if BDNF dependent.

(1) "As suggested, we performed qPCR and observed that 1 month-old KO midbrain and cortex express lower levels of Dbn1 as compared to WT brains (Figure 5G). This result is in agreement with the western blot data (Figure 5H)."

There is no Fig 5H? 5F? In 5F effect sizes are exaggerated with axes not crossing zero. There is a 10% reduction in mRNA (normally >1 or 2 fold changes would be considered biologically important?). This isn't much change, and should be presented as such. 1 month old WB in G are much more convincing of a reduction of drebrin levels, but what brain region is this from?

We apologize for the error in the rebuttal, where we incorrectly referred to figure 5G (the correct is 5F), while what we called 5H is instead 5G. We checked the labeling in the manuscript text and it is correct.

Following the Reviewer’s important suggestion, we rescaled all plots to start at zero. Although some differences appear relatively modest, they are statistically significant. Importantly, all brains used for qPCR analyses (N = 6 per genotype) were obtained from independent mice. In addition, independent cohorts of mice were used for spine morphology analyses (N = 3 per genotype), TEM analyses (N = 4), and western blot experiments (N = 3). Thus, the overall sample size across approaches is substantial.

WB are from whole brain, now indicated in the figure legend.

All blots are underpowered, especially given what appears to be an age dependent loss of drebrin in both genotypes beset by high variability

(i) Western blots looking at pSer935 and pRab8 (pan Rab) in Dbn1 WT and knockout brains.

"As reported and quantified in Figure 2I, we observed a significant decrease in pSer935 and a trend decrease in pRab8 in Dbn1 KO brains. This finding supports the notion that Drebrin forms a complex with LRRK2 that is important for its activity, e.g. upon BDNF stimulation."

Non-sig data in Fig2I/H and especially Fig5G are important data but hard to interpret because the experiment is underpowered. I am surprised the authors designed studies on an n=3 western blot.

For fig 2 this is a problem if they wish to correlate LRRK2 activity with drebrin. The KO have a clear 50% decrease in LRRK2 pS935 but no change to pRab8(pan).

As discussed above, we increased the sample size by 7 additional mice per genotype (total of 10 brains analyzed).

Why not look at Rab10, and certainly redo with a higher n than three. Of special confusion is the observation that the WT with the highest drebrin levels, is the animal with the lowest pS935 & pRab

As discussed above neither pRab8 nor pRab10 returned convincing results in the new round of western blots. We acknowledge that future experiments should explore the phosphorylation levels of Rab12 which is emerging as a more reliable readout of LRRK2 kinase activity in the brain.

(ii) "Reverse co-immunoprecipitation of YFP-drebrin full-length, N-terminal domain (1-256 aa) and Cterminal domain (256-649 aa) (plasmids kindly received from Professor Phillip R. Gordon-Weeks, Worth et al., J Cell Biol, 2013) with Flag-LRRK2 co-expressed in HEK293T cells. As shown in supplementary Fig. S2C, we confirm that YFP-drebrin binds LRRK2, with the N terminal region of drebrin appearing to be the major contributor to this interaction"

CoIP with drebrin (and fragments) is very convincing.

We thank the Reviewer for his/her comment/feedback

Ephys data, presentation, and response to review is weak.

We reanalyzed the data as suggested by the Reviewer and reviewed the text and interpretation.

**Reviewer #2 (Recommendations for the authors):**

p. 12, last paragraph. "sealing" should be "ceiling"

We corrected the misspelled word
